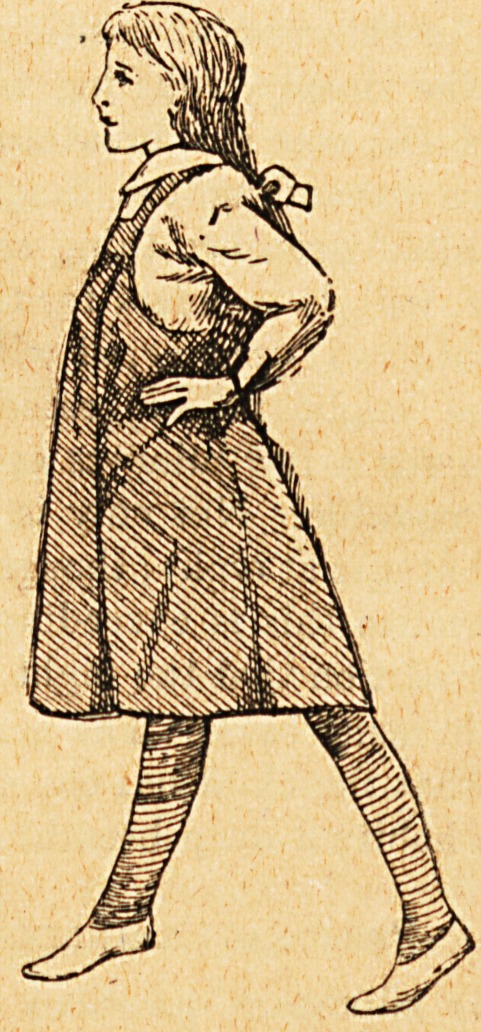# The Hospital. Nursing Section

**Published:** 1906-10-13

**Authors:** 


### The Hospital.

### Nursing Section.

Contributions for "The Hospital," should be addressed to the Editor, "The Hospital" Nursing Section, 28 & 29 Southampton Street, Strand, London, W.C.

No. 1,047.—Vol. XLI. SATURDAY, OCTOBER 13, 1906.

### Notes on News from the Nursing World.

### THE NURSES' HOSTEL.

We are glad to hear on authority that last Friday's Board Meeting may lead to a settlement which will satisfy all parties. We have therefore decided, in the hope of contributing to this result, to hold over for a week the further communications we have received, so as to give time for the result of the deliberations of the Board to be made known and in justice to the claims of Miss Hulme.

### THE MIDWIVES BOARD AND THE PRIVY COUNCIL.

It will be seen by our report of the last meeting of the Central Midwives Board that they do not see their way to acquiesce in the amendments of the rules suggested by the Privy Council with respect to training in Poor-law institutions. These amendments have, however, been put forward after due deliberation, and it may be presumed that the Privy Council has both the power and the will to insist upon their introduction. In spite of a long diatribe in a lay paper denouncing, in sweeping terms, the nursing in Poor-law institutions, and the persistent attempt which is being made in another quarter to prevent their recognition as training schools for midwives, we think that the Local Government Board will not lightly submit to the continuation of a policy seriously inimical to interests which they are obliged as a department to protect.

### NURSING TYPHUS IN IRELAND.

It can scarcely be believed that the state of affairs described in the "Incident in a Nurse's Life," which appears on another page, could have prevailed in the present century. As a matter of fact, however, our contributor writes from her own experience during the months of June and July in the present year. According to her testimony, it is only when an epidemic of typhus—which occurs yearly—breaks out that the Guardians by whom she was employed recognise the mischief, and as soon as the outbreak is over they settle down again to the old routine, and make none of the urgently needed alterations. It is obviously imperative in the interests of humanity that lavatories should be attached to each ward, and that there should be more than one tap of water to supply the whole of the building. Nurses in the pursuit of their avocation cannot, and do not, wish to avoid certain risks. But there are some to which they ought not to be exposed; and though our contributor passed through her perilous ordeal in safety, it is inconceivable that, after her revelations, similar dangers should have to be encountered in Kerry next summer or on any future occasion.

### AN ENGLISH MATRON FOR A FRENCH HOSPITAL.

We learn with pleasure that Miss A. F. Davidson, sister of Job Ward in Guy's Hospital, has been appointed matron of L'Hopital Ruffi, Nimes, Gard, France. This hospital, which was founded in 1360 by M. Ruffi and holds over 360 beds, 50 of which are set apart for the soldiers, is partly under Government, and hitherto the nursing of the sick has been done by nuns. Miss Davidson, who therefore inaugurates a very important new departure, entered Guy's in November 1893, and has, since her training, been on the private staff of the Nursing Institution, night superintendent, and sister of Martha, Cornelius, and Job Wards. She also went out to nurse the wounded in the Turko-Greek and South African wars. Miss Davidson will have as assistant matron Miss Helen Lambert, who completed her training at Guy's Hospital in 1899, and has since been sister of Bright, the paying ward. They will take up their duties on Wednesday next.

### GUY'S HOSPITAL.

The departure of Miss Tippetts and other sisters and nurses from Guy's Hospital to the Mayo Hospital, Lahore—to which we referred last week—has rendered necessary several new appointments. Miss Mabel A. Chittock, who completed her training in 1903, and has since been sister at Charing Cross Hospital, assistant home sister and night superintendent at Guy's, has been selected to succeed Miss Tippetts as assistant matron. Miss Frances A. Sheldon, who completed her training in 1903, and has since been sister-in-charge of the wardmaids, has been appointed to succeed Miss Newton as sister of Lydia Ward. Mrs. I. F. Hill, who completed her training in 1905, and has since been theatre sister and night superintendent, has been appointed to succeed Miss Morrah as sister of Charity Ward. Miss Edith Lear, who completed her training in 1905, and has since been instructress in the Preliminary Training School, has been appointed to succeed Miss Webb—home sister—as sister of Astley Cooper Ward; and Miss L. E. Jolley, who has just completed her training, has been appointed instructress in the Preliminary Training School. Miss Eleanor Macfarlane, who completed her training in 1904, and has since been on the private nursing staff, has been appointed to succeed Miss Davidson, who has been chosen as matron of L'Hopital Ruffi, Nimes, as sister of Job Ward; and Miss E. Windener, who completed her training in 1903, and has since been on the private nursing staff and instructress in the Preliminary

Training School, has been appointed to succeed Miss Lambert—who accompanies Miss Davidson as assistant matron of L'Hopital Ruffi to France—as sister of Bright Ward. Miss A. H. Clarke, sister of the housekeeping department, has been appointed instructress in the Preliminary Training School; and Miss Jessie Orr, sister of Patience Ward, has been appointed sister of the housekeeping department. Miss Lissie Bonser, who completed her training in 1905, and has since been on the private nursing staff, has been appointed to succeed Miss Orr as sister of Patience Ward. Miss Elsie Fraser, who completed her training in 1905, has been appointed medical night superintendent.

### THE MIDDLESEX HOSPITAL.

The usual series of medical and surgical lectures to nurses by members of the visiting staff at the Middlesex Hospital will begin on October 18. Classes in elementary nursing will also be held for the junior probationers by the assistant matron. During the past year thirty probationers have been selected for trial from about four times that number of applicants. The new Nurses' Home, with its spacious dining-hall and nurses' sitting-rooms and pleasant, bright bedrooms, is increasingly felt to be a source of comfort and health to the nurses. Seven additional good rooms have been acquired this year, thus rendering it possible to accommodate a great number of wardmaids who materially aid the nurses in various duties which had hitherto fallen to the latter's share, particularly in the evening work of the wards. Scrubbers in the wards are now practically abolished. The annual holidays are nearly over, and it is a gratifying fact that the temporary duties of the matron of the cancer wing, the sisters, the home sister, and night superintendent have been very satisfactorily discharged by members of the permanent nursing staff, some of them private nurses and others senior staff nurses.

### THE TESTIMONIAL TO MISS MEDILL.

It has been intimated to the subscribers to the testimonial to Miss Medill that the presentation will take place in the Board Room of St. Mary's Hospital, Paddington, on Thursday, November 1, at 5.30 p.m. The testimonial will consist of a silver bowl, an album of autographs of the sisters and nurses past and present who have joined in the testimonial, an album containing their photographs, and a purse of gold.

### WEST LONDON HOSPITAL.

In March last two adjoining houses were taken as a Nurses' Home for the West London Hospital, and this has been the means of enabling a good many other improvements to be effected. What were the nurses' quarters have now been converted into a children's ward, of which there had been great need, the babies having been in the same ward as some adult patients. They now reign supreme in a bright, airy ward of their own, which provides accommodation for twenty beds. The children's ward first came into use in July, and the ward thus released is in process of transformation into a much needed out-patients' hall, with all necessary partitions. Hitherto, for lack of room, all out-patients have had to wait in the general hall of the hospital, and the only out-patients' department has been a tiny slip of a room. The latter has been converted into a waiting-room.

### NOTTINGHAM GENERAL HOSPITAL.

There are two points in the interview between our Commissioner and the matron of Nottingham General Hospital, reported on another page, which will be noted with special satisfaction by some of our readers. Miss Knight, instead of objecting to probationers who have received previous training in a children's hospital, considers it a recommendation, always, of course, providing that the candidate produces satisfactory references from the institution where she was trained. The advocates of the use of outdoor uniform will be glad to know that the matron of Nottingham General Hospital not only regards it with favour, as both desirable and convenient, but sets the example of habitually wearing it herself. In these circumstances the nurses attached to the institution are naturally expected to pursue the same course; and it does not appear that they offer any objection.

### FEVER NURSES AND THE PENSION FUND.

An address on the subject of the Royal National Pension Fund was delivered by the Secretary, Mr. Louis H. M. Dick, on Monday evening last to a meeting of nurses at the Northern Fever Hospital, Winchmore Hill, under the presidency of the medical superintendent, Dr. C. E. Matthews. There was a large attendance of nurses, including a contingent from the Isolation Hospital, Willesden. At the conclusion of the meeting several nurses joined the fund.

### THE QUESTION OF CUBICLES.

The question of further accommodation for the nursing staff is engaging the attention of the Mile End and the Preston Boards of Guardians. In both cases it has been proposed "to improve the sleeping accommodation for nurses" by providing cubicles. No final decision has, however, been arrived at in either. As one of the Mile End Guardians protests against the nurses being allowed during the winter "to sleep like dogs in a kennel or rats in a hole," it must be necessary to take action without delay; and the need of further provision for the nurses at Fullwood Workhouse is admitted by some of the Preston Guardians to be pressing. But we strongly urge both Boards not to expend the money of the ratepayers in the erection of cubicles. If they decide, as we hope they will decide, to try and make things more comfortable for their nurses, why not provide them with separate bedrooms? On every ground the latter are preferable to cubicles, and the cost need not be materially greater.

### DISCHARGE OF THE MATRON OF A CONVALESCENT HOME.

One of our correspondents last week alluded to the dismissal of the matron of a convalescent home near Bath under circumstances somewhat similar to those attending that of Miss Hulme. We have since been informed that the whole of the inmates of the institution in question, 27 in number, have signed a letter addressed to the authorities expressing their deep regret at the retirement of the matron and their entire satisfaction with the manner in which she has discharged her duties.

### TRAINING AT BRIGHTON POOR-LAW INFIRMARY.

The proposal to train probationers at Brighton Poor-law Infirmary has been rejected by 16 votes to 14. The report of a special committee on the question was discussed at length at the last meeting of the Guardians. The committee recommended, subject to the sanction of the Local Government Board, that a resident medical officer be appointed to the workhouse; that steps be taken with a view to the erection on land adjoining the West Infirmaries of quarters for a medical officer, the superintendent nurse, and 50 nurses; and that a small operating-theatre be erected with a covered passage connecting the West Infirmaries. Mr. Lader strongly supported the adoption of the report, and urged that it was a great drawback to the institution that, under the existing system, probationers in the infirmary could not qualify for the position of superintendent nurse at the expiration of their time because there is no resident medical officer. The objections to carrying out the recommendations were based upon the ground that the new building would cost a large sum of money. This is true, but the standard of nursing cannot be raised to meet the requirements of the times without essential structural additions; and in the meantime young women intending to be nurses will recognise the serious disadvantages of entering as probationers at Brighton Poor-law Infirmary.

### IRISH NURSES' ASSOCIATION.

The first of a series of lectures which have been arranged for the winter months in connection with the Irish Nurses' Association was given on Friday evening last by Mr. J. De C. Wheeler, M.D., F.R.C.S.I., on "The Functions of the Brain and Spinal Cord." The lecture was most interesting and keenly appreciated by the large number of members who were present. A vote of thanks was proposed by Sister Wright, seconded by Sister Maconachie, passed by acclamation, and conveyed to the lecturer by Mrs. Kildare Tracey, who presided.

### NURSES IN THE FOREIGN MISSION FIELD.

A feature of the reunion of the Nurses' Missionary League last week at the Church Missionary House in Salisbury Square was an address from Miss Theodora Fox, who is starting this month to take up work at Gosha Hospital, Bangalore. Miss Fox, who was trained at the Middlesex Hospital, suggested that nurses at home could be of great help to nurses in isolated places if they would correspond with the latter and tell them of interesting cases they are nursing, new methods and treatments, and give them any other information which would enable them to keep up with the times. She was sure that in return the isolated workers would supply intelligence about their work, their interesting cases, and their special needs. In addition to Miss Fox, Miss Hilda Cooke, who was trained at Edinburgh Royal Infirmary, Miss Winifred Gordon and Miss Eva Pearson, who were both trained at the Mildmay Mission Hospital, are leaving England shortly for service in the foreign mission field.

### SCHOLARSHIPS FOR STUDENTS OF MIDWIFERY.

We are informed that in January 1907 the London County Council will award not more than six scholarships to students in midwifery. Candidates must reside within the County of London and be between 24 and 40 years of age. The value of each scholarship will be £25, and the course of training will extend over a period of six months. Forms of application may be obtained from the Executive Officer, Education Department, Victoria Embankment, W.C., and must be returned to the same address not later than Saturday, October 20, 1906.

### SICKNESS OR OLD AGE.

At a recent meeting of the Royal Victoria Trained Nurses' Association papers were read suggesting that the moneys of the benevolent fund should be applied to the use of nurses in the event of sickness. Miss Glover, however, urged that the working nurse, if she falls ill, has practically the benefit of free nursing and free medical treatment; that medicine is supplied to her at cost price, and that she is cared for in the convalescent home until her restoration to health is assured. The prevailing feeling was that the interest in the benevolent fund should be devoted to meet cases of distress among old and worn-out nurses. There is no doubt that, generally speaking, provision for nurses in sickness is as easy as it is often difficult for nurses no longer able to work.

### THE VISITING NURSE AT CAMBRIDGE.

The new visiting nurse under the Cambridge Corporation has been appointed. That the post has a good many attractions may be gathered from the fact that there were upwards of 60 applications, the successful candidate being Miss Alice Dearden. This lady was one of four who attended before the sub-committee, and, in addition to being a local candidate, she was considered to possess most excellent qualifications.

### OUR CLOTHING DISTRIBUTION.

The necessity of repeating our appeal to our readers in the interests of our clothing distribution at Christmas has been brought home to us by a letter from a correspondent, who, although she saw our first intimation, confesses that she has forgotten the address to which the articles should be sent. We therefore reiterate the statement that all parcels must be sent to the Editor, 28 and 29 Southampton Street, Strand, London, W.C., with "Clothing Distribution" marked on the outside. We hope by the end of the present month to be able to announce the receipt of a long list of contributions.

### SHORT ITEMS.

The St. Andrew's Ambulance Association and Corps, whose headquarters are at Glasgow, have issued the first number of a promising official monthly organ under the title of *The Red Cross and Ambulance News.*

### The Nursing Outlook.

"From magnanimity, all fears above;
From nobler recompense, above applause,
Which owes to man's short outlook all its charm."

### CHILDREN, DISEASE AND CRUELTY.

In every community consisting of poor people there are a number of children, and even some adults, affected by diseases which demand prompt treatment that they frequently fail to get. The experience of every worker amongst the sick in hospitals, and especially of members of the medical profession, is that large numbers of such cases of neglect, due often to carelessness, are reduced to a condition which renders their state almost hopeless, when they ultimately come to the hospital. The causes which underlie this state of affairs and the remedy by which alone it can be cured must form the subject of a separate article. At the moment we wish to bring out clearly the relations that exist between children, disease, and cruelty which is caused by neglect in the case of these little ones.

An immense amount of good, entailing the removal and saving of great suffering in children which might otherwise have ruined their lives, has been effected by the Invalid Children's Aid Association. This Association, by dividing densely populated neighbourhoods into districts, getting into communication with all the authorities, and others, who have a knowledge of the poor and their condition, combined with a system of visitation of cases in their own homes, has accomplished a splendid work. It appeals to all humane people, and especially to women-workers, and for this reason numbers in its ranks some of the most kindly and sympathetic souls of both sexes. They continuously labour and are continuously affording evidence of the value of the services rendered by the results which they achieve. Their methods are permissive, not compulsory. They approach the parents or guardians of a child who needs treatment or help and use their influence to obtain consent to the child's removal to a hospital or to such other conditions as may be best calculated to restore it to health. If they fail in effecting the best result they achieve gradual success very often by means of the district nurse, and their own personal devotion to the service of each little sufferer.

It happens sometimes, however, that permissive measures are not successful in moving the parents or guardians to a sense of their duty when cases of great difficulty arise which may result in the permanent injury or death of the child. The Society for the Prevention of Cruelty to Children has mainly confined itself in the past to cases which the Act had first in view, which include ill-treatment, neglect, and exposure of children in a manner likely to cause necessary suffering or injury to their health. The question of medical advice or its absence stands on a different footing. A man or woman is not obliged, if seriously ill, to call in medical advice in their own case at all. Neither transgresses any law by refraining from doing so. But those who have charge of children under sixteen years of age are bound to send for medical aid when it is necessary. If parents or guardians do not call in skilled advice and the child dies as a result they are guilty of manslaughter, although they may have treated the child with every care and attention in every other respect. A conviction for manslaughter in such cases is certain when it can be shown that the action of the parent or guardian having charge of the child, by omitting to call in a doctor, has had the effect of shortening the child's life.

Cases of neglect resulting in death stand on a different footing, and the law on the subject is not at present as clear as we could wish. The neglect to take a child to the hospital or to call in medical advice may be due to a mistaken idea of kindness on the part of the parent for the child, or even to great affection. In one such case which was fully reported in the *Times*, the Society for the Prevention of Cruelty to Children summoned the mother for neglecting her son by declining to have an operation performed upon him which the medical evidence showed was essential to the boy's interests. The boy was suffering from necrosis of the left thigh-bone. He had already been operated upon four times. The child was shown to have been well cared for by the mother, who had built up his general condition by intelligent treatment, but the medical testimony was that the boy ought to be placed under the care of a surgeon, and that an operation should be performed. The refusal of the mother rendered this step impossible, and the Society sought a conviction to enable them to obtain the custody of the child, and so have the operation performed. The magistrate demurred to the action of the Society on the ground that it wanted to punish the mother because she would not obey, and dismissed the case on the further ground that in his opinion the Society began the proceedings too quickly. The Society's answer, which is reasonable, is that after a long period of supervision, repeated warnings and the ultimate production of a medical certificate by the examining practitioner, they issued a summons in the interests of the child. No final conclusion as to the state of the law can be gathered from the case, but it appears probable that should the Society have a similar case and produce the evidence of a specialist a decision may be reached which may finally settle the point. Meanwhile the interests of the parents rather than of the children are likely to prevail in similar cases.

### The Care and Nursing of the Insane.

By Percy J. Baily, M.B., C.M.Edin., Medical Superintendent of Hanwell Asylum.

### II.—NURSING THE SICK.

### 1. General Remarks.

Sick nursing is an art which belongs peculiarly to women because its proper performance depends very largely upon the possession of that gentleness and sympathy which are essentially feminine characteristics. But in order that a woman may be a good nurse certain personal qualities must be added. Some of these are attributes which constitute what we know as character and are more or less inherent, others must be acquired by education and all can only be perfected and developed by special training. It is not our intention to discuss here the personal qualities which go to make a good nurse, but it is right to point out what some of the most essential of these are.

A nurse must seek to gain the confidence of her patient and all those traits of character which command the confidence and admiration of others, are those which a nurse should strive to cultivate. She must above all things be truthful and conscientious. No human being is perfect, and nurses, like everyone else, are apt to make mistakes; she whose errors are the fewest, and who, when they do occur, has sufficient force of character to own them, is the one who gains and most deserves the confidence alike of the patient, the patient's friends, and the doctor.

In many cases of sickness the success of the treatment depends almost entirely upon the accuracy with which the nurse carries out the doctor's instructions. This is almost entirely the case in acute and curable conditions, and in cases where recovery cannot be expected the length of the patient's life may be said to depend also upon the care bestowed in the nursing.

Good nursing is really dependent upon the observance of a number of minute details, and no one can be a good nurse who does not thoroughly appreciate the fact that no detail, however small and apparently insignificant, is too trivial to demand careful attention.

It must ever be remembered that when nursing becomes necessary it is because the patient is passing through a period of suffering and great trial, and the nurse should never lose sight of the fact that her whole object is to minister to the patient and to increase as far as possible his comfort during this time, to foresee and if possible avoid all sources of danger and discomfort, and to endeavour to know and supply his wants before he is able to recognise them for himself.

When dealing with insane patients it may often be impossible to carry out the details of nursing with the same degree of precision as is the case with those whose minds are normal, but nevertheless such should be the aim of the nurse, and it frequently happens that during the course of many acute diseases the mental state of the patient may be so modified as to bring this aim quite within reach.

Everything must be done by the nurse with as little fuss and as little noise as possible, but this must not be carried to an excess. It is a great mistake, for instance, for a nurse or anyone else to walk about the sick-room on tiptoe, or to carry on any conversation in whispers. As a rule this is only irritating to the patient, who will not be disturbed by the ordinary movements of those about him unless they are exceptionally clumsy, nor by the voice unless it be unnecessarily loud or high-pitched.

### 2. Definitions.

When an organ or tissue performs its functions in such a way as not to depart from the standard of health it is said to be normal. Any departure from this standard gives rise to a condition which is abnormal.

When during the course of a disease the anatomical structure of an organ is altered the disease is spoken of as an organic disease. Symptoms of disease often arise on account of the functions of an organ being performed in an abnormal manner without there being any discoverable structural alteration in the anatomical arrangements of the organ. Such disease is spoken of as functional disease.

A disease is said to be acute when its symptoms are severe and its course rapid, but when it becomes established and runs a slow and lingering course it is said to be chronic.

### 3. The Sick-room or Ward.

In asylums and other institutions of a similar kind certain wards are set apart for the treatment of those who are physically ill or infirm and no choice is left to the nurse in their selection. But every nurse ought to know what are the essential requisites of a sick-room or ward as to its aspect, size, equipment, etc., and the following remarks are intended to suggest an ideal which should be approached as nearly as possible. The sick-room should be one which has to the greatest attainable degree an air of cheerfulness, with an abundant supply of fresh air and sunlight, these being two of the very best purifying agents. If the room is being selected for a private case, and therefore is situated in a private house, a room on the ground floor should be chosen as being more handy to the source of supplies, except in cases of infectious disease where isolation is necessary; for such cases the room should be at the top of the house. The windows should look towards the south and west, and should be capable of being thrown wide open if necessary both at the top and bottom. In a large ward where there are several windows these ought to be arranged on opposite sides and should be in the east and west walls. Unfortunately, the rules of the commissioners in lunacy do not permit the windows in the wards of lunatic asylums in this country to open more than five inches either at the top or bottom. Each window should be provided with a roller blind made of dark green material. The object of the blind is to darken the ward if need be; if they be made of light material this object becomes unattainable. An exception should perhaps be made when the windows are overlooked and it is necessary to use the blinds for purposes of privacy.

The more spacious the sick-room is the better is it suited for its purpose, because a large room is more easily ventilated without producing appreciable draughts than is a small one.

The walls should be perfectly smooth, and should be painted rather than papered, since painted walls are more easily kept clean. When they are painted they should be wiped over with a damp cloth or sponge once or twice a week. If papered the paper should be perfectly plain and entirely devoid of pattern. There should in any case be no lines or squares or phantastic shapes which may produce a bewildering effect upon the wandering mind of the patient. Whether painted or papered, they should be of some subdued and unobtrusive colour.

The floor should be of wood blocks, or should be entirely covered with linoleum, and in any case should be waxed and polished so that it may be kept clean without having to be scrubbed. Scrubbing is old fashioned, and as a means of cleanliness is deceptive and dangerous, since the dirt is simply washed up into a thick mud, which settles in the crevices between the boards. When the floor is not polished, however, it is, of course, a necessary evil, but it should be resorted to as seldom as is consistent with a decent appearance. The polished floor should be cleaned at least once or twice a day by rubbing it over with a damp house-flannel tied for convenience over the end of a soft broom. By this means the dust can be removed without its being simply swept up into the air until it again finds a resting-place in some other part of the room, which invariably takes place in the ordinary process of sweeping. The floor should afterwards be dry-rubbed. Even a floor composed of ordinary boards may be treated every day in this manner, and thus the evil of scrubbing be postponed. On no account should any fluff be allowed to gather under the beds or in the corners of the wards. Spread about the floor there should be a few mats or strips of carpet for the patient to stand on when getting out of bed, and also to deaden the sound caused by the movements of the nurse and others in the room.

The furniture ought to be simple but good. No unnecessary article of furniture should find a place in the sick-room, since it would only occupy air space which should be left vacant. There should be no heavy hangings either about the bed or the windows. The window curtains, without which no room can be cheerful, should be of some light washable material.

*(To be continued.)*

### The Nurses' Clinic.

### OVARIAN CYST CASES.

A patient suffering from ovarian cyst may feel very little inconvenience at the beginning, and the first symptom she usually complains of is a gradually increasing sense of fulness and swelling of the abdomen. She may suffer from constipation or frequency of micturition through the cyst coming in contact with other organs.

The preparation of the patient for ovariotomy will be the same as for most other abdominal operations. The patient will require to have an aperient and a bath early the day previous to operation, the surface of the abdomen must be thoroughly shaved and cleansed from grease, etc., with soap and water, turpentine, methylated spirit or ether, and washed with an antiseptic solution, a compress of lint saturated with the same solution being placed on the surface prepared, covered with a pad of gamgee and firmly fixed with a bandage. Surgical cleanliness must be observed by the nurse during the process of preparation.

An enema of soap and water must be given the next morning, and the patient may have a light breakfast four to five hours before operation. The nurse must see that the bladder is emptied by a catheter immediately before the patient is taken to the operating theatre.

The patient should wear a gamgee jacket round her chest, covered by a short dressing jacket fastened at the back and long stockings. When on the operating table her chest must be covered with a small blanket, another being placed over her legs. She will probably be operated on in the Trendelenberg position. The principal instruments etc. required will be: scalpel, scissors, retractors, twelve pair artery forceps, dissecting forceps, pedicle clamp forceps, pedicle needle, curved cutting needles, large and small, intestinal needle, needle holding forceps, ovariotomy trocar and cannula with long piece of tubing attached, large basin to catch fluid from cyst (which ought to be measured), silk and catgut ligatures, silkworm gut sutures, sterilised abdominal and ordinary towels, mackintoshes, small swabs of gauze, long pieces of gauze with tapes attached for abdominal sponges (which must be counted before and after operation), pads of gamgee and many tailed or ordinary bandages.

The nurse must have plenty of sterile water in readiness, hot and cold, also antiseptic solution; saline solution ʒj to the pint may also be called for to wash out the abdomen after the cyst has been removed. After operation the patient must be kept warm in bed with blankets and hot-water bottles, a pillow to support the knees and a cradle to lessen the weight of the bed-clothes. When retching or vomiting occurs the nurse must place her hand firmly on the abdomen to save strain on the stitches.

Very little, if anything, ought to be given to drink for the first twenty-four hours after operation, the mouth may be washed occasionally with water and a pint of tepid water may be given per rectum if the thirst is very great.

If after the first twenty-four hours the sickness has passed off, fluids—milk and water or kali, chicken soup, beef tea, barley water tea, etc., may be given, at first in small quantities, afterwards increasing to three or four ounces hourly.

An aperient is usually given on the third night after operation, and when the bowels have acted freely, a little light solid diet may be given, beginning with bread and butter or toast, milk pudding, etc., and gradually increasing until the patient is on ordinary light diet.

A four-hourly chart of temperature, pulse and respiration should be kept for the first week after operation, the urine must be measured and charted. The patient ought to be encouraged to pass urine herself from the first; failing this the catheter will be necessary, but not oftener than eight-hourly, great care and cleanliness being observed by the nurse at each passing. Pain caused by flatulence may be relieved by the use of the rectal tube.

These cases generally suffer a great deal of pain for the first few hours after operation, and where the surgeon

THE NURSES' CLINIC—*Continued.*

objects to giving morphia the nurse may be able to soothe the patient by a little tactful sympathy and assurance that matters will soon improve. The shock is never so great as in hysterectomy cases, but in the case of a delicate patient, who may be much collapsed, the foot of the bed may be raised and saline or brandy enemata given if ordered by the doctor in charge.

The same care and precautions must be taken as in all abdominal operations, the patient being carefully watched and all abnormal symptoms reported without delay.

The surgeon usually removes the sutures about the end of a fortnight, and the patient will probably be allowed to get up in about three weeks from the operation if everything has gone well.

### Incidents in a Nurse's Life.

### AN EPIDEMIC OF TYPHUS.

A private nurse attached to an institution can very rarely call herself free. Last June, as I had returned the evening before from a very trying mental case, I indulged in the luxury of breakfast in bed and an extra hour's rest while looking over my letters. As the morning advanced I congratulated myself that no case would turn up for me that day, and, as I was feeling very much "run down," decided to go for a short sea-trip to steady my nerves.

No sooner was I dressed and ready to start than a message arrived for me to proceed at once to the most remote part of Kerry to relieve a nurse in charge of the Fever Hospital who had been attacked by typhus fever. I was aware that an epidemic was raging in that district. It is by far the poorest and most congested part of the country, and every year this dread visitation passes over the land, often sweeping whole families off the face of the earth, and generally carrying off a nurse or two also. This was a must undesirable case, and being rather "unstrung," for the only time during my nursing career I felt the nearest approach to fear. My first impulse was to tell matron that I was quite unequal to a fever case just then, and to ask her to get another nurse to go in my place. But, on referring to her note, I found that she had no other nurse in, and that she was compelled to send me. Then putting aside my selfish fears, I thought of my poor friend laid up in a strange place, and how welcome the sight of a familiar face would be to her in her weak condition, and, going down on my knees, I placed myself under the protection of the Divine Providence Who watches and cares for us all.

I found that there were barely twenty minutes to catch the only train for that place; so, hastily changing into my uniform, I started off, and reached the station in the very nick of time.

Never shall I forget that journey. It was by far the dreariest I had ever taken. It seemed also the longest, for once we left the beaten track and branched off from the Dublin line the whole aspect of the country changed. Mountains surrounded us. The air became quite chilly, and what was but a mist before became a heavy downpour now. The last passenger departed from the carriage, leaving me in icy isolation, with my far from cheerful thoughts for company during the remainder of my journey. For one thing, the fact that I had not touched food since breakfast and saw no prospect of anything coming at 7 p.m. set "Little Mary" clamouring for some kind attention and lowered my spirits accordingly.

The train still crawled slowly on, stopping occasionally at every little landmark on the way. How I strained my eyes to see if by any chance I could procure a cup of tea at one of these resting stages. But alas! the language of the country even was changed, the very names of the different little stations were in an unknown tongue, so with very doubtful philosophy I resigned myself to the inevitable. As I proceeded my morbid feelings of the morning returned with renewed force. I tried to banish sad thoughts by reading, but to no purpose. The carriage appeared to me full of those poor nurses who had come this same journey on the same mission and who had never returned. They kept constantly beckoning me to follow them; some were quite familiar to me, others I had never known. But all looked bright and perfectly happy, and seemed delighted at the prospect of my joining them.

I awoke with a start (for, of course, I had been dozing) and looked round; but the carriage was, as before, perfectly empty. I could not shake off the effects of the dream—the presentiment became almost a conviction that I was going to certain death, and every mile I travelled seemed to bring me nearer my end. It was a horrible sensation. I tried to find solace in the thought that I should be found at my post doing my duty to the last. But my mind would persist in dwelling on the loved ones I had left behind, whom I felt I had seen for the last time. I am generally in the very best of spirits starting off on a case; but I must confess that typhus at all times had a certain horror for me, for every nurse I knew who ever took a case of it in Kerry succumbed to the disease.

Again that awful train stopped. I had been disappointed so often that this time I kept my seat, and not until I discovered that it had come to stay did I venture to alight and look round. But my troubles were ending, for almost the first being I discerned on the platform was a young lady in nurse's uniform, who was evidently looking out for me. Though strangers to each other, I cannot describe the feeling of relief and comfort which I experienced on meeting that girl. I was cold, tired, ill, hungry—she knew exactly all about it without my telling her. She had done that journey herself and suffered similarly, so her sympathy was genuine.

She took me at once to the only hotel the place boasted of, where she had ordered dinner for me; but after my long fast (well over twelve hours now) the very sight of food was too much for me. I could not eat a morsel, but was very grateful for the warmth and luxury of a strong cup of tea.

We then proceeded to our destination—the Union; my new friend was the permanent nurse in the infirmary there. She introduced me to the sister, who at once noticed how ill I was looking and went off in search of the doctor. When he came he pronounced me quite unfit for duty that night; so sister procured the services of a "Gamp" for the night and saw me comfortably in bed after giving me some nice hot milk.

That rest saved my life, for I feel certain that if I had been obliged to go on duty after that journey I should never have got over it. As it was, after a refreshing sleep and breakfast I felt fit for anything again. So I went to visit the field of my labours where my time would now be spent, and found I had in all, both male and female, seventeen patients, some wildly delirious, a few in a comatose condition, one or two at the point of death, and all the others more or less bad; in fact, I saw at a glance that I should have my hands very full indeed, for there was not a soul to be had to give the smallest help to a nurse, as no salary would induce anyone to go as ward's maid to the Fever Hospital.

The hospital consisted of four good-sized wards, two for male, two for female patients, a moderate-sized kitchen, and a small apartment called by courtesy the "doctor's room," which I was to use as a dining and sitting-room.

On making a few inquiries of the sister in charge, I found to my horror and amazement that the water supply was very defective, the whole hospital being dependent on one solitary tap, and that a very indifferent one indeed. But, worse than a thousand times, I discovered that sanitation was unheard of; in the whole of that building there was not such a thing as a single lavatory. The sister assured me that since they had charge of the place they had been constantly agitating and bringing this matter before the Board of Guardians, but so far nothing had been done to improve the condition of affairs.

Now for my presentiment. No wonder any poor nurse coming to this district under such circumstances fell a victim. How could she possibly expect to escape infection when the authorities did absolutely nothing to check the spread of the fever; indeed, their utter carelessness and neglect only fostered it?

I knew the worst now, and almost gave myself up for lost. There was nothing for it but to do all I could to relieve the poor sufferers, speak a cheering word to them when consciousness returned, and try to alleviate their miseries in every possible way. In a word, to do my duty faithfully till my turn came.

At 9 P.M. I came on duty, read the report, went my round of the wards, gave out medicines and stimulants, and sponged three patients whose temperatures were very high. A few had to have hypodermic injections, there were drinks to be given all round and several other little attentions which all fever patients require, so that by the time one round was completed it was nearly time to begin all over again.

However, the "Gamp" was induced to stay, for which I was honestly glad, as any company was better than none. She was a woman of the lower orders, quite illiterate, slovenly to the last degree, and, like all of her class, suffering from an inordinate affection for spirits and its usual accompaniment—snuff. In fact, the very sight of her set me off sneezing without a moment's notice—she literally reeked of snuff and whisky. But, bad as she was, it was comforting to feel some sane person was about, although my very soul revolted at the sound of her loud grating voice. Finding she knew most of the male patients, I gave her charge of them, superintending her, of course, myself. I found her very attentive indeed, and kind in her own rough way. Nearly all spoke in the Irish language, so she was useful also in that way as interpreter, for I did not understand it.

But all I could do would not teach my ancient "probationer" the use of disinfectants. "Wisha, miss," she would say to me, "shure the favor is all the will of God, and them stuffs in the bottles will never take it away." Another thing I had great difficulty about was changing the patients' bed and body linen. I had to be very strict with her over this. But with all my vigilance she was sometimes too clever for me.

On one occasion going into the kitchen unexpectedly I found the fire-place surrounded by sheets and shirts spread out on chairs, a dense vapour arising from them, and the odour—well, the imagination must supply that. By a great effort I got her to tell me what this display really meant. She was drying the articles which she had removed from some of the worst patients who perspired profusely. This was done so as not to give the extra work to the laundry-maid, who happened to be a friend of hers. Naturally I was very indignant, and ordered her to remove them instantly to the wash-house, saturating them myself with carbolic in the meantime. Very reluctantly she obeyed me, grumbling all the time, and, of course, thinking it nonsense for me to give so much trouble over such trivial matters.

Well, I managed to live through three weeks at this place, and as no fresh cases were arriving and those who survived were now convalescent, the doctor decided that he could dispense with the services of the "trained" nurse. I was charmed when I heard this, and cheerfully resigned my post in favour of my "pro., Mrs. Gamp."

Next morning I was off by the first train, thanking God for my escape and hoping never to go near that pestilential swamp again, or at any rate while the present state of things exists. How different everything looked along the line! I could scarcely believe it was the same route I had travelled such a short time before. The day was beautiful, the mountains were bathed in brilliant sunshine, the air was redolent with the exquisite perfume of new-mown hay, all nature was at its best. Even the strong nasal twang of the American tourists ceased to jar when they joined us at Killarney. In fact everything seemed in harmony with my own happy thoughts, for here I was, alive and well, speeding back to those dear to me, whom I feared at one time I should never see again.

### A District Nurse and a Case of Advanced Phthisis.

The patient whom the district nurse is called upon to see after is presumably of the lower middle class, too poor to afford a private nurse, and yet not too abjectly destitute to be unable to have things moderately decent and comfortable. Were this not so it would be the duty of the district nurse to advise the removal of the patient to the workhouse infirmary.

If the nurse is given the choice of a room for her patient she should choose the largest and sunniest one in the house, also, if possible, one that contained two windows. There should be no carpets on the floor. Oil-cloth or linoleum would be the best covering, or failing that plain scrubbed boards. The walls should be white-washed, but, if papered, the paper would have to be removed on the death of the patient. There should be no superfluous drapery or articles of furniture about, but to redeem the room from bareness lace curtains might be put to the windows, a few bright-coloured prints nailed on the walls, and some nice plants or flowers placed about. All dust-harbouring bric-a-brac, photographs, etc., should be carefully removed. The patient's bed should be a small iron one of the usual hospital pattern. If he is going in for the modified open-air treatment his bed should be put close under one of the windows, and the window either taken completely out of the frame, or else the lower part always kept wide open. Of course the doctor's directions would be followed implicitly as regards this. The other window should be open top and bottom continually. In foggy, extremely cold, or very wet weather the window by the patient's bed would need to be closed, but in all weathers the other one should be left open a few inches at the top. The patient must be protected from draughts by an easily-moved, large-sized screen of some sort. There must be a fireplace in the room and, except on very hot days, a bright

### A DISTRICT NURSE AND A CASE OF ADVANCED PHTHISIS—*continued.*

though not large, fire should be kept burning day and night, not only on account of the ventilation and warmth, but as a means of cheering the patient.

The vessel into which he expectorates must be lined with three layers of thick news or brown paper, and half-filled with a reliable disinfectant. Cyllin 1 in 16 is absolutely a sterilising disinfectant against the bacillus of tuberculosis in its most resisting form. The paper and contents must be taken out and burned at least three times a day. The bed-pan in use must also be frequently rinsed with the above powerful disinfectant and the floor sterilised by washing every day with it. All plates, cups and other utensils used by the patient must be kept for him only. He must not use ordinary handkerchiefs, but pieces of rag or soft paper, which should be burned directly they are soiled. His shirts and bed-linen will need to be soaked in disinfectant, or else washed and boiled by themselves. His diet, of course, will be ordered by the medical man, but in a case of very advanced phthisis one cannot lay down any hard and fast rules as to feeding. It is as well to let the patient take what he will and tempt his appetite as much as possible. Also, if he sometimes expresses a desire to have his windows closed, it will do him less harm to shut them temporarily than to have a fuss with him on the subject. Great attention must be paid to the prevention of bed-sores. However if the patient is washed all over quickly night and morning with very hot water and plenty of soap, his back and all prominences well rubbed with spirits and powder, and clean dry shirts put on him, it will not be likely that he will suffer from soreness of any part, especially if a water or air pillow is used. Notice must be taken of the action of the bowels and all tendency to diarrhœa reported to the physician. If there be any hæmoptysis, however slight, the friends should be instructed to keep a small supply of ice in the house, and should be told how to use it. Finally, they should be warned that when the patient is irritable and exacting it is a symptom of his complaint, so that they need not unduly distress themselves and imagine that their kind offices are not appreciated.

### The Nurses of Nottingham General Hospital.

### INTERVIEW WITH THE MATRON. BY OUR COMMISSIONER.

The situation of the Nottingham General Hospital is not only unique because it stands on an eminence from which the whole of the city may be seen; it has also historic associations which are valued by the citizens. At the corner of St. James's Street, close to the building, is Newstead House, which was occupied by the poet Byron from 1798 to 1799; while on the other side of the road, just below the

A Ward in Nottingham General Hospital.

new circular wards, there is an inscription to the effect that "On a mound about 80 yards to the rear of this tablet Charles I. raised his standard August 25, 1642." I must confess, however, that on the occasion of my visit to the hospital last month, I was less impressed by the tablets recording these interesting facts than by the most admirably carved figure of The Good Shepherd in a niche at the entrance just inside the gates. Nothing could be more suggestive of the purpose of the institution, of which the inhabitants of Nottingham, from its generous and considerate Chairman to the humblest of operatives, are justly proud. Waiting for a minute while my card was sent in to the matron, Miss Gertrude Knight, I noticed the handsome entrance hall and the appearance of brightness. The latter pervades the whole of the building, which is remarkable for the absence of anything resembling dark corners. As the matron informed me soon after I was shown into her charming suite of rooms, the additions and alterations which have been in progress for several years are now just finished, and one effect has been to secure the maximum of light and sunshine obtainable. The great addition is, of course, the Jubilee Extension. which was finished six years ago, and I spent some time in the circular wards in this wing. They are remarkably lofty and airy, with windows all round.

"Even in the hottest weather this year," said the matron, "these wards were comfortable."

"You have everything up to date here," I observed, noticing the teak floor, the glazed brick walls and other modern appointments.

#### The Home.

Having been through a few of the wards, seen some of the patients in the balconies enjoying the air, expressed my appreciation of the beautiful chapel and the singularly well-cared-for mortuary, glanced at the modern cooking apparatus (which is one of the features of the fine new kitchens), and inspected the Nurses' Home, I began to ask questions about the nursing.

"How many does the Home accommodate?" I inquired, as we stood on the terrace above the delightful garden which belongs to the nurses.

"Sixty. The Home was formed out of four houses which are divided into two groups, and as you see, the whole of the rooms are particularly lofty."

"Does each nurse have a room to herself?"

"With just a few exceptions, which are due to the fact that the rooms would not lend themselves to alteration. These," added the matron, as we went on, "accommodate two nurses."

"They are certainly unusually large and airy rooms. I notice that in the single rooms also there is a fire-place."

"Yes, in every room. The corridors are heated with pipes; but I think that fire-places are desirable both for purposes of heating and ventilation."

"Is there an adequate supply of bath-rooms?"

"One to every five nurses, or the proportion exceeds this if the night staff are taken into account. The quarters of the night staff are quite shut off. As to other provision, there is a spacious sitting-room with piano, and a quiet room for study."

"Also I see that the nurses make use of an excellent croquet-lawn."

"Two of the night staff are playing just now in their off-duty time. But they all regard the private garden as a boon."

### The Training and Age.

"Sixty does not, I gather, represent the full strength of the staff?"

"No; with nine sisters there are sixty-eight. There is also the assistant matron and home sister, who, by the way,

Nurses' Sitting-room at Nottingham Hospital.

was trained at King's College. Most of the sisters, however, were trained here, and some of them have been in the hospital a good many years. There is a housekeeper, but she is not a trained nurse, and I do not find it any disadvantage that she is not."

"How long has the period of training been three years?"

"It was initiated fourteen years ago, when I came. I was trained at St. Bartholomew's Hospital, and was matron of the Royal Victoria Eye and Ear Hospital. Dublin, and matron of the Adelaide Hospital before my appointment to Nottingham."

"At what age are probationers admitted?"

"The minimum age is twenty-one and the maximum thirty-five. I consider about twenty-three the most desirable; but I think that institutions which do not take probationers under that age often lose the most suitable candidates."

"Do you object to previous training in a children's hospital?"

"Not in the least, if a candidate produces good recommendations from the hospital where she was trained. In fact in such circumstances I regard it as an advantage."

### Paying Probationers and Salaries.

"You take paying probationers, I believe?"

"A few for not less than three months for a payment of thirteen guineas. They are amenable to precisely the same regulations as the others, and the only reason we receive them is that they may have the opportunity of obtaining a knowledge of practical nursing, which is often useful at home."

"Do the ordinary probationers pay an entrance fee?"

"Yes; but they are paid a salary of £6 the first year, and the entrance fee is only ten guineas. The second year the salary is £10, and the third year £16."

"For what term do they come on trial?"

"Two months. It is not possible to judge of their fitness in less. Of course, in addition to practical instruction in the wards, they attend lectures by the medical staff and the matron; and in their third year they are examined by the medical staff. A course of massage is given yearly."

### Outdoor Uniform.

"Is there much difference between the hours of the sisters and the probationers?"

"Very little. The probationers rise at 6, breakfast at 6.30, enter the wards at 7, dine at 1, are off duty alternate days from 3 to 6, have tea at 5, supper 9.15, retire at 10. The sisters have a month's holiday, the first year probationer two weeks, and the others three weeks."

"Are all meals served in the hospital?"

"Yes. The sisters have no sitting-room in the Home, but each one has her own room off the ward.

"What about uniform?

"Material for dresses, caps, and aprons is supplied, and the wearing of uniform out of doors is expected. It consists of a blue cloak and bonnet. I always wear it myself, and regard it as both desirable and convenient."

"How many nurses are on duty in a ward?"

"Three day and one night nurse, in addition to the sister. There are now 242 beds, an increase of nearly a hundred since the new wing was opened. Each of the circular wards contains eighteen beds, and there are also two small wards on each 'flat.'"

### The Work in the Wards.

"Are the beds generally all occupied?"

"Yes; we nearly always work at high pressure, and the medical and surgical experience gained is very valuable. We have every modern appliance in the theatre. As soon as we can we draft the patients on to our Convalescent Home, The Cedars, Sherwood, about three miles from Nottingham. It has thirty-eight beds and is under the same Chairman.

Nurses' Home and Private Garden, Nottingham Hospital.

"Do you take any other than general cases?"

"No. As you saw, there are two wards with nine cots for children, and a further number of children pass through the wards. But we receive no fever cases except typhoid, and they are strictly isolated, as well as the nurses who attend on the patients."

"Your out-patient department seems a very important one?"

"It is. The number of cases during the year average

THE NURSES OF NOTTINGHAM GENERAL HOSPITAL—*continued.*

2,174. A sister is in charge, and she has always the assistance of a nurse."

"Then I conclude that your probationers who cannot become sisters easily get good appointments after they have completed their training?"

"We keep on a limited number as staff nurses, and pay very strongly in favour of State registration. I advocate it because, in my opinion, it would help to give the nursing profession its proper status."

Leagues and Registration.

"Do you favour the formation of Nurses' Leagues?"

"I do, although we have not yet got one here, and I am very strongly in favour of State registration. I advocate it because, in my opinion, it would help to give the nursing profession its proper status."

Subsequently the matron showed me the rooms provided for the domestic staff in the new wing, which are far better than the quarters of nurses in some institutions, each commodious cubicle containing a fixture wardrobe, chest of drawers, and wash-stand with marble top. In fact the great idea of the Chairman—to whom the hospital owes many things and whose interest is unflagging—is that the patients, the nurses, and the household should be comfortable; and, so far as the nurses are concerned, it may fairly be said that their lives appear to have fallen in goodly pleasant places.

### Midwifery in Sumatra.

For fourteen years I had been a Red Cross nurse in a German Hospital. Then I left for a bigger field of work, and I was also influenced by the fact that some relations of mine had married and gone out to Sumatra as missionaries.

My brother-in-law fetched me from Sibuga, where I landed from Europe, admiring intensely the lovely cliffs and gorgeous foliage of Sumatra, backed by the mountains with their beautiful woods and high white tops. When I had finally said good-bye to the boat I began to grasp the fact that I was in another country, but did not fully realise how different Asia is from Europe until I discovered that there was no need to grumble at the railways, the slow omnibuses, or the smelling motor-cars, because there were no such things! I had never ridden in my life, not even on a donkey for a penny ride, and lo, I found my brother-in-law waiting for me with two horses, one for himself and another with a side-saddle—for me! Of course I was not going to let a "mere man" think a woman was afraid, and so I was hoisted on to my steed, and off we went. At the time it felt very peculiar, but I soon got used to the necessity, and now I enjoy riding immensely. The journey from Sibuga inland was very beautiful; it reminded me very much of the Swiss Alps, except that every now and then in the winding road one caught glorious glimpses of the sea. The plant-world is wonderful, very luxurious, too; we saw ferns thirty and forty yards high, no end of different palms, and the route was a succession of charming dells covered with lovely ferns and flowers, and high mountains with white shining glistening tops, and glaciers, more like a brilliant dream than grim reality.

Next day, with my brother-in-law, who is a missionary, we rode for four and a half hours and spent the night in a "passentenhaus"—traveller's rest—which the Dutch Government has built for officials travelling through the country. The next morning we got up at four a.m., so as to proceed in the cool and escape the great heat of the day and were in the saddle until half-past nine. When we arrived at our destination I stayed with my sister and brother-in-law for a few days, intending to go straight to the hospital of which I was to take charge when sufficiently rested. But after two days a doctor I was to be under at the hospital came to tell me that he had a woman patient right on the east coast (we were on the west), who was expecting her first child, and who was very ill. He had promised to go, but unexpectedly had two serious cases which he could not leave, and so he asked if I would go. Naturally, I promised, being glad to be of use so soon, and also delighted at the rare opportunity of seeing quite a different part of the country.

I now found that eleven "brothers" were also going to look at some land suitable for building a new Mission-station, so I could accompany them. A boat was procured from the Dutch Government, and so with the "eleven" and my brother-in-law, who was to accompany me across the country, we started off next morning at seven a.m. and had a lovely time at first, and I thoroughly enjoyed Nature's luxurious beauty. But at mid-day the weather suddenly changed, and became windy and then stormy. The waves got quite rough and very high; we very often could not see the little Government tug that was towing us, as we were in a deep valley of water, and the tug above us, hidden by the sea-horses' foam. We were in great danger of being swamped several times and expected our last moment had arrived, but after three hours' delay we arrived safely. I scarcely realised our danger, as I felt fearfully sea-sick and generally wretched, a feeling not lessened by knowing that I was the only woman in the boat. It is dark in Sumatra all the year round at six p.m. We ought to have landed at 3.30 p.m., but owing to the storm we did not get there until half-past seven, when the light had quite gone, and the natives could only help us with great difficulty and danger. We were thankful to be on land once more—but how different here from comfortable Europe! After the trying crossing, the fatigue, and sea-sickness, etc., I would have given anything for a European room, but we had to find a place to rest in for the night. A sort of camp was made for the men at the Punditen's (native pastor) house, and I was taken to a room. Everyone travelling in Sumatra carries provisions, his own mattress and blankets. Fortunately my brother-in-law had seen that I had taken all needed for my use. We naturally had to lie on the floor, as the "Bataker" do not possess bedsteads, etc.—even the Radjats (tribal chiefs or kings) have none, nor chairs—but when very polite the natives provide rice-sacks for Europeans to sit upon! I need scarcely say that, in spite of being dog-tired, I slept hardly at all, for it was too lively in my room!

Early next morning off we had to go again. The young missionaries and I parted, they for the very heart of the country, and I for the east coast. Now I found an unexpected difficulty which was rather funny at first. Of course the people where I was to go expected the doctor and sent a horse for him, consequently there was no side saddle! However, "in for a penny, in for a pound," I thought, so I told my brother-in-law I could manage quite well, though inwardly I could not help laughing at the thought of what the sisters and nurses in Germany would say if they could have seen me riding like a man.

My brother-in-law borrowed a horse and off we went happily enough. Soon we came to a very steep hill, which we had to climb, then through very tall grass so high that it reached over our heads, in spite of us being on horseback. Later we rode right through huge maize-fields. The horses are wonderful, the guides lead them over the most difficult places, and where very steep the animals draw their hind legs under them and slide down. At first I did not expect to be alive at the bottom, but the brave little fellows are so clever and quite used to the hard work, and, I daresay, smiled inwardly at me! By way of a change we forded a river, and once we had to get off our horses, as the banks were too steep to ride down, and there were huge pointed stones. Once I hurt my foot very badly, but as I was not a tourist or travelling for pleasure, but was going to help a sick woman, I had no time for rest, and it did me no harm. Arriving at the other side we again passed through high grass which had caught on fire, and the flames burnt over our heads, so literally we had been through fire and water. And to crown it all, in a few minutes we were caught in a real tropical shower of rain, so that, in spite of waterproofs, unbrellas, etc., we were drenched through and through, but we kept on, and after the rain soon got dry through the great heat. We came to Rajah at 5 P.M. at last, alive and well, but tired. Luckily, I enjoyed a thorough night's rest, and was glad I had come, for the next night, after much danger, the patient became a mother at 10 P.M. of a boy. Now she is perfectly well and strong again, though at first we feared she would not recover. In three weeks' time I was able to leave again, and should have loved to take the little fellow with me, but the parents objected! They were so kind and grateful, which fully made up for all the hardships of the journey, and they told me that I was only the second white woman who had ever been there.

Then I went to Si-Sorang, where I often helped in the Hutas (villages) and often had most difficult cases. Once I had to ride for two hours and a half, and found a woman whose dead child had been born three days before, and who was nearly dying through neglect—and dirt. But, strange to say, she is quite well again. Taking into account the dirt and squalor and the peculiar ways, recovery seemed almost impossible. But, as they say, *Allah is great.*

The natives have absolutely no idea of medical cleanliness. Imagine a hen-coop at home—one has to climb steps exactly like those in a hen-house. Then one enters a dark, gloomy place with undescribable odours of dirt, heat, etc. There are no windows, but after getting a little used to the gloom and stench one sees numerous figures and hears a great noise with crying and weeping. After a little time, I need hardly say that the room is cleared, as in Europe. The great secret of nursing there is to give the neighbours something to do, if only to fetch a glass of water, then they disappear for a time at least. The patient generally lies on an "cnak" (straw mattress), dirty and uncomfortable. One does one's best, but often the sufferers will not allow a white woman to come near, fearing she will impart the evil spirit to the child to be born. Sometimes, in consequence, the woman and the child die through want of a little skilful midwifery.

I have charge of the midwifery school and wards. At first it was most difficult to teach the "Bataken," as they had no feeling of responsibility and were most unconscientious—but now I understand them better and like them very much; as soon as they gain confidence they are very docile and eager to learn. When not working I find that the life here is interesting; most of the Europeans are clever, and have more interests than the average people at home. We have literary meetings, but I especially enjoy the musical evenings, as some of the people play exquisitely and sing well. Moreover, missionaries often stop here on the way back from China and Europe, all of which gives the charm of variety to the life of a nurse away from her own people.

### Lady Roberts' Nurses.

### A PIONEER MOVEMENT.

Numerous inquiries are made from time to time as to the work done in India by Lady Roberts' Nursing Staff. So far back as 1886, Lady Roberts—who was then residing in India, and, as wife of the Commander-in-Chief, took a great interest in all that concerned the troops, and especially the sick officers and men—drew up a scheme for supplying skilled nursing to the Military Hospitals. In India, where there are so many serious cases of enteric, dysentery, and heat stroke, requiring the best of care and nursing, it seemed to her very essential that nurses thoroughly well trained should be supplied. Hitherto, the soldiers had been dependent on the "tender mercies" of their comrades or the orderly on duty, who, though they might do their best to look after and attend them, were not trained and had no knowledge of the requirements of a sick officer or soldier; and too often many lives had been endangered or lost. Greatly, as one does and should admire and respect the hospital orderlies, who have done noble work in taking care of their sick and sometimes dying comrades, when there have been no others to help, yet nursing is essentially a woman's work, and the patience and concentration of purpose required to make a good nurse, is more usually found in a woman's character than a man's. This, I venture to think, was the opinion of Lady Roberts.

#### The First Step.

And as an experiment she sent out several fully trained lady nuises, who, as pioneers, started working in the military hospitals at Lucknow, Bareilly, Quetta, Meerut, and other places where a large number of troops were stationed, and these pioneer nurses had no light task to perform, getting suitable articles for their patients and introducing new and better methods for the comfort of those under their care. So well did the experiment succeed, and so much were the services of Lady Roberts' nurses appreciated that a few years later the Government undertook to provide and send out a staff of nurses to carry on and extend the work so well begun, under the superintendence of Miss C. Loch. This was the beginning of that service, now entitled Queen Alexandra's Imperial Military Nursing Service in India.

#### Nurses for Officers Only.

When these Government nurses arrived Lady Roberts withdrew her nurses from the station hospitals, to work more especially in the officers' hospitals at Murree and Kasauli, and to nurse free of charge officers who were ill in stations where nurses could not be procured. I venture to maintain that there are few stations in the Punjab and North-West Frontier which have not at some time or other received the services of some of Lady Roberts' nurses, of whom it has been said:

> "They are ever ready and ever true
> To the toils and tasks they have to do."

During the Tirah campaign three of them were employed at the Base Hospitals at West Ridge and at Nowshera, and did

LADY ROBERTS' NURSES—*continued.*

excellent work. Very pretty and unique badges are worn by these nurses, designed by Lady Aileen Roberts, the elder daughter of Earl Roberts.

The Nurse's Badge.

The badge presented to the lady superintendent is of gold, and those presented to the nursing sisters are of silver. The design is a St. Andrew's Cross, within a circle, with bars according to length of service. On the 18th of December, 1902, Miss A. J. Weighall, the present superintendent, was presented with the order of the Royal Red Cross by His Majesty King Edward at Buckingham Palace.

The Hospital at Murree.

The officers' hospital at Murree is a very useful institution. Here officers are sent who require skilful doctoring and careful nursing. Each officer has a separate ward and home comforts are, as far as possible, provided. The lady superintendent is assisted by two fully trained lady nurses, and soldier orderlies also help to nurse and look after the patients. Of the 300 or more officers who have been admitted to the Murree Hospital between 70 and 80 have been enteric cases, mostly young fellows between the ages of twenty and thirty, just out from England, whose fresh young blood is so ready to catch any poisonous germs that may be hovering about. The officers have said how well and how carefully they are looked after, and how comfortable are their wards. But I understand that more ground is required to enlarge and improve the building and compound. The medical officer in charge belongs to the Royal Army Medical Corps, but there is a civil ward attached, and the patients admitted to this ward are attended by the civil surgeon. This ward was opened in 1902 and the money collected for the building of it was due to the efforts of Col. J. Montgomery, C.B., a former Commissioner of Murree and Rawul Pindi. He simply asked the members of the Punjab Commission to subscribe, which they readily did, and the deed was done. This ward has been a great boon to young officers of the Indian Civil Service, who come up to Murree ill, and have no other place to go.

The Nurses Themselves.

The nurses belonging to Lady Roberts' Nursing Service are personally selected by herself, and are of an education and social status to fit them to enter a service where they rank next to officers. They engage for, and are expected to serve, five years, but in case of unsuitability or other causes their services can be dispensed with on their being given six months' notice. In case of proved disobedience to orders or neglect of rules they can be dismissed at once. Nurses can in like manner end their term of service by giving six months' notice. The lady superintendent's salary is 250 rs. a month, the nursing sister's 200 rs. and 150 rs. A rupee is equal to one shilling and four pence. During the summer the nurses live free of all charges for food, servants, fuel and lights.

The Outfit.

Nurses receive an outfit consisting of grey dress faced with royal blue, cloak and bonnet and caps and aprons. Uniform must be worn by the lady superintendent and nurses on all occasions, indoors and out, except when on leave. In hot weather white dresses with blue collars and cuffs and for an evening entertainment white silk. The lady superintendent is responsible for the entire management of the hospital, both as to domestic economy and nursing, and she arranges the hours at which the nursing sisters are to be on duty, and assists in the nursing at all times when necessary. While Lady Roberts is anxious that her nursing staff should have as much wholesome recreation as possible consistent with their duties in the shape of outdoor amusements, and friendly parties, she does not allow them to go to balls or entertainments entailing late hours, which she considers detrimental to their health and strength, usually sufficiently heavily taxed by their nursing duties, and unfits them in mood and temper for attendance on the sick.

### How to Strengthen Delicate Children.

BY A SISTER.

It would be difficult to say how many children are suffering from a weak back, either the beginning of serious spinal trouble or the weakness of general debility. It is easy to distinguish the stooping gait or the slovenly carriage of the embryonic curvature, so that no observant nurse would be accused of unnecessarily making a diagnosis when the plain facts make it so evident. It is often the privilege of a nurse to be able to give advice in matters of hygiene without encroaching on the preserves of a medical man. She may even prepare the way for him to finally cure the trouble. So often, when one is known to be a nurse, especially in "districts," the anxious mother will say, "I wish you would tell me what to do for my little girl's back; she is always complaining of a tired feeling and an aching back." Then comes the chance of the common-sense hygienic nurse to expound her views on proper diet, plenty of fresh air and baths, and enough rest, especially in the middle of the day between school hours. The sensible mother, though perhaps deplorably poor, will do her best to follow out the simple instructions for the benefit of the child's general health and the probable prevention of a crooked spine. Where, however, there is distinct curvature present the nurse must, of course, tell the mother that the doctor must see and prescribe for the child. The following hints may be of use to those who desire to show some of these poor people their error—whether through negligence or ignorance—in acting contrary to the laws of nature and violating the laws of health.

Judicious Feeding.

First, with regard to diet, the poorest person can generally obtain good wholesome bread and butter and milk. If the mother is led to see that this is sufficient diet—with the addition of fresh fruit, syrup, porridge, broth, and a few simple inexpensive things of similar value as foodstuff given sometimes to vary the meals a little—a great point will have been gained; and if she is also made to understand that the sticky messes sold in cheap shops as sweetmeats and also unwholesome cakes must be avoided at all costs, hygiene will have scored again. It is the children who are irregularly and insufficiently fed who clamour for such unsuitable fare; therefore it should be suggested most emphatically that a proper meal should be given at regular hours, as far as possible, to prevent this. One finds the poor half-starved, undersized child when admitted to hospital for one or another malady absolutely refusing to touch milk or milk puddings. This is the result of their early training, or, to be more correct, their early neglect. Fortunately, with tact and perseverance, one often comes off victorious, and is rewarded by seeing "Tommy" or "Mary" eagerly devouring the nicely-made milk pudding at last; and further reward is vouchsafed the successful nurse when the little one goes home healthy and a striking contrast to the victim of bad feeding on admission.

### Fresh Air.

Fresh air is the next thing of vital importance; and, thanks to the County Councils of to-day, the public gardens and parks are accessible to all. A few minutes' walk or tram will bring the children to a miniature country. The best thing for the tired back is to lie flat for some time each day; the open, and fresh air is, of course, to be preferred. While in this restful position the child should be taught to breathe deeply, inhaling as much fresh air as possible through the nose and breathing out slowly through the mouth, thus expanding the lungs and flattening the back. Ten minutes' deep breathing night and morning, or even once a day, will do wonders for the narrow little chest and poking shoulders if persevered with every day. A few simple exercises might be suggested which the mother should see done for a few minutes each day, these also in the open air if possible. Not only is fresh air so essential in the daytime but also at night; though to make many of the poorer classes realise this is exceedingly hard. The clinging to the old tradition that the night air is harmful is still very tenacious in many cases. The mother should be got to promise to see that the child has the window open at night and to practise habitual deep breathing.

### Personal Cleanliness.

When the two important questions of diet and fresh air have been more or less assimilated, the equally important one of personal cleanliness should be introduced. Again, thanks to the Councils, in these better days we have every facility for the bath; but there will always be difficulties for the unwilling participator. The child will probably be too young to be trusted to go to her bath alone, and the mother is frequently of the "no time" type. The nurse should then suggest that the ablution be performed at home —if the outside public bath becomes an impossibility—at least twice a week. The apparatus may be of the most simple order—in fact, the ordinary family wash-tub can be utilised. Water and soap are so cheap that one dares to become emphatic and declare that there is no reason why even the poorest should not take their daily bath and become quite as devoted to it as they are to their meals. If the mother has been won over on the side of hygiene on the foregoing points she will probably not be difficult to manage here; and if she has proved difficult of conquest heretofore, maybe now she will decide to be on the side of cleanliness. As to the rest a delicate child should have, it is difficult to decide if the patient belongs to parents who can neither give nor afford supervision; but she should be encouraged to lie on her back for an hour or two, especially if she goes to school and sits over a desk in a cramped or one-sided position, for although the fast prevailing hygienic spirit has spread to schoolroom furniture, crooked backs also still prevail, and demand a reaction from the stooping uniform posture of the little scribes.

These suggestions may be quite impracticable for some mothers to carry out; but although they may be unable to do so to the letter, yet they will, no doubt, be led to see the importance of them and the benefit to be gained by their adoption where and when possible. The process of uprooting ancient traditions is always slow, and one can only hope for sure progress, if not rapid. It will be felt that the persuasion, the explanation, the suggestion, or whatever other method may have been used, has not been in vain if on any one point the mother and child are actuated to become devotees of the goddess "Hygeia."

### Simple Exercises.

The accompanying sketches will give some idea of a few simple exercises which would be beneficial to a child suffering from general weakness and the usual narrow chest. To teach her to hold herself correctly and upright will perhaps be a matter of time, for habits are not altered in a day. From these illustrations other exercises may be evolved, as may be seen from the following remarks.

This shows the position to be taken first of all. Hands on hips, shoulders well back, head up, heels together, knees stretched, and the whole body perfectly upright. This is a good position to take for breathing exercises, drawing in a deep breath through the nose and exhaling through the mouth.

Here the child is shown with arms fully stretched, during which she inhales deeply and simultaneously. On bringing the arms down to the sides she exhales slowly. When she can do this well she may also add foot exercise and rise on her toes as the arms are raised, coming down slowly on

### HOW TO STRENGTHEN DELICATE CHILDREN—*continued.*

her heels as the arms come down, breathing deeply all the time.

This is another good exercise for chest expansion. The hands are at first loosely clasped behind. At the word of command—counting one, two, briskly—the palms meet and the shoulders are thrown well back. This may also be done with deep breathing. Other arm exercises for expansion of the chest may suggest themselves, but one must always keep this end in view so that no exercises bringing the hands together in front may be countenanced: "Shoulder width apart" must be the rule for the arms when stretched up, down, or in front. One or two more exercises may prove useful. (*a*) The patient takes the first position, arms stretched level with shoulder (one), forearm flexed, hands brought forward, palms down, fingers close together and stretched, thumb touching chest (two); forearms, arms again extended smartly (three); repeat ten or twelve times. (*b*) Patient in same position, the arms may be thrown back and round, but never meeting in front, starting and finishing on a level with shoulders, then down. All these movements should commence gradually until the muscles become accustomed to the extra strain. The patient should not be overtired, so that it is best to begin with three or four times each exercise, working up to twelve, twenty, or more each movement.

Now the patient is in fall-out position. Starting with the left foot, and, as all foot and leg exercises should, from the first position. She goes smartly forward keeping the right foot firm, throwing back the head and shoulders at the same time, then regaining the first position. To do this without moving the firm foot requires some practising, and it is excellent as a balancing exercise, bringing all the flexors and extensors of leg into play, as well as influencing the abdominal muscles to some extent. It may be done with alternate legs or six times each leg and so on.

This is the tip-toe exercise, another good one for balance and exercise of the leg muscles, and indeed of the whole body, because the first position has to be maintained throughout. It should be done very smartly and kept up for two or three minutes if not too tiring; deep breathing should be encouraged all the time. To exercise the trunk the patient may take up the first position, then bending forward, keeping the whole of the leg muscles well stretched and still, she should swing the body to the left, back as far as possible, and round to the right, and repeat several times. These exercises should not be used for any curvature or deformity; a medical man must always decide first whether they will be suitable for any such case, but, generally speaking, for a child merely stooping and weakly, these movements will prove most beneficial. The child in the illustrations has much improved in general health since the represented exercises started some months ago.

### Meeting of the Central Midwives Board.

The Central Midwives Board met, for the first time since the summer vacation, on Thursday last week, at Caxton House, Dr. Champneys taking the chair. Miss Paget, Miss Wilson, Mrs. Latter, Mr. Fordham, and Mr. Parker Young were also present.

### The August Examination.

The minutes of the two meetings in July were signed and the financial statement read. The Secretary's report on the August examination showed that out of a total of 245 candidates 192 passed the examination and 53 failed. Of these candidates 148 represented the various training schools in London, and 124 passed; 27 out of 33 from the provincial schools in England, five out of seven in Scotland, and five out of six in Ireland passed; while out of a total of 50 candidates who had received private tuition 31 satisfied the examiners.

### An Appeal against the Decision of the Board.

The next item on the agenda was the Secretary's report on the appeal to the High Court, brought by Ita Feldmann, against the decision of the Board ordering the removal of her name from the roll and the cancelling of her certificate. Owing to a lack of evidence at the hearing of the case it was adjourned till November. Meanwhile it appears that Mrs. Feldmann is not prohibited from practising as a midwife. On the motion of Mr. Fordham, seconded by Miss Wilson, the Secretary was instructed to take the necessary steps to see that the Board is represented in the proceedings in the High Court.

### Recommendations of the Penal Cases Committee.

The report of the Penal Cases Committee was then dealt with. A letter was read from the Clerk of the Cheshire County Council, reporting the finding by the local supervising authority of a *prima facie* case of negligence and misconduct against Hannah Hibbert. It appeared that in a case at which she was attending the child died soon after birth, and the midwife, not having summoned medical aid, was censured by the coroner. The committee recommended that Hannah Hibbert be cautioned as to the necessity of strictly observing the rules in future, and this was agreed to.

In the case of Deborah Mabel Goddard, who was accused of malpractice, negligence, and misconduct, it appeared that she had allowed a child, whose birth she had attended, to be placed out to nurse within 24 hours of birth. This fact had come under the notice of the Infant Life Protection authorities. The recommendation of the committee, that the attention of D. M. Goddard be called to the fact that she appears to have neglected her duty under Rule E, 11 (which makes "the midwife responsible . . . for securing the comfort and proper dieting of the mother *and child* during the lying-in period"), and that she be cautioned as to its strict observance in future, was agreed to.

With regard to the other cases, Adela Flora Zeifert, Jane Baker Rimmer, and Alice Luty, the recommendation of the committee, that they should be cautioned as to their future conduct, was agreed to. Twenty-three women were cited to appear before the Board, that the charges against them might be fully gone into. November 8 was the date fixed for a certain number of these to be heard.

### The Privy Council and the Board.

In the report of the Standing Committee (which met on September 27) a letter, referred to the committee by the Board, was read from the Clerk of the Council, suggesting certain amendments to the rules as revised by the Board. The committee recommended that the Privy Council should be informed :—

"1. That the Board do not see their way to acquiescing in the amendments suggested by the Privy Council with respect to the Poor-law institutions.

"2. That the resolutions referring to the number of cases qualifying institutions or teachers for approval by the Board were framed merely for their own guidance, and were of a tentative nature only. The Board did not wish them to be stereotyped in any way, and do not consider that they are 'Rules' within the meaning of the Act requiring formal approval by the Privy Council.

"3. That the suggestion that a new Rule should be made imposing upon midwives the duty of notifying to the local supervising authority every birth occurring in their practice has already been considered by the Board, who thereupon resolved that in their opinion the matter was one for legislation, and not for a Rule of the Board."

The Chairman explained that with regard to the second of these clauses the committee had thought it advisable to make a fixed number of not less than 60 cases per annum a condition of a teacher being approved by the Board, but this proposal, as he said, was merely tentative, and could be altered as they might find necessary. Replying to the objections raised by Mr. Parker Young, Miss Paget pointed out that the number of cases was not the only thing to be considered. In institutions where there were only one or two pupils it was difficult, if not impossible, to arrange systematic courses of lectures; and the Chairman added that the lack of an atmosphere of obstetric study was detrimental to the training of our future midwives. After some further discussion, the recommendation was carried unanimously.

### Correspondence.

A letter was read from the Secretary of the Royal Commission on the Poor-law and Relief of Distress, asking the Board to nominate a witness to give evidence as to the attitude of the Board with regard to recognising Poor-law institutions as training schools for midwives. The committee recommended the Board to pass the following resolution : "That, inasmuch as the Rules of the Board are at present under revision, the Board considers the time inopportune for nominating a witness." This was passed without further comment.

A letter was read from Dr. Hope, Medical Officer of Health for Liverpool, calling attention to a circular issued by a certified midwife, advertising herself as a herbalist. The committee recommended "that Dr. Hope be informed that the Board possesses no power to interfere under the circumstances stated." The Chairman added that people were apt to forget that midwives were not doctors, and what would undoubtedly be derogatory to a medical man might not be so to a midwife.

Letters were read from Dr. G. S. Clarkson and Dr. T. Robinson, Medical Officers of Health for Leicestershire, asking for information as to the causes of failure of three candidates at the August examination. The committee recommended "that Dr. Clarkson and Dr. Robinson be informed that it is contrary to the practice of the Board to give reasons for the rejection of candidates at its examinations." The Chairman explained that they would be unable to do so, as the Board did not examine; it only appointed examiners. The inquiry was a reasonable one, as the medical men in question wished to give extra instruction to their pupils in those matters in which there had been failure.

### Certificates of Training.

A resolution was carried that under Section C of the Rules the following application for approval as an institution for the training of midwives be granted : Government Maternity Hospital, Madras. The applications of the Allbless Hospital, Bombay, and the Leeds Maternity Home were refused. Recommended by the committee, the following applications for approval as a teacher under Rule C, 1, were granted : J. Cromie, L.R.C.P.&S.(Ed.), L.M.; A. E. Weld, captain R.A.M.C. The following similar applications were refused : F. Edmunds, M.R.C.S., L.R.C.P.; A. Thompson, M.R.C.S., L.R.C.P.; T. G. Vawdrey, M.R.C.S., L.R.C.P.; a motion on the part of Mr. Parker Young in favour of Mr. Thompson's application being lost.

Edith Mary Hepplewhite, 10 Cambridge Street, Leicester, was approved for the purpose of signing Forms 3 and 4. A similar application from Agnes Harriett Withers, Military Families' Hospital, Curragh Camp, Ireland, was refused, and another from Sarah Adelaide Glasgow, 264 Grosvenor Road, Belfast, was postponed for further inquiries.

The date of the next meeting of the Board is fixed for November 1. The Standing Committee will meet on Tuesday, October 23, at 4 p.m.

### Everybody's Opinion.

[Correspondence on all subjects is invited, but we cannot in any way be responsible for the opinions expressed by our correspondents. No communication can be entertained if the name and address of the correspondent are not given as a guarantee of good faith, but not necessarily for publication. All correspondents should write on one side of the paper only.]

### THE NURSES' HOSTEL.

We have received a number of letters respecting the situation at the Nurses' Hostel, but, as will be seen from a "Note" on another page, we have decided to hold over all allusions to the subject in the hope that before next week the Board will have arranged a settlement satisfactory to all parties.

### "SO-CALLED TRAINED NURSES' ASSOCIATIONS."

Nurse M. H. writes: I want to bring before the notice of all trained nurses the question, Do they consider it fair for women to set up so-called "Trained Nurses' Associations" and then employ nursemaids, lady-helps, and the like? The general rule is to pay 5s. down, and the managers say that they will do their best; but you hear no more and lose your 5s. If only some gentlewomen would set up a genuine registered nursing association I feel certain that they would succeed.

### CANCER CASES.

The Secretary of the Cancer Hospital, Fulham Road, S.W., writes: In answer to Inquiry No. 7 under your "Notes and Queries" column last week you are good enough to mention this hospital as admitting cancer cases. May I supplement, for your future use, the further information you give with regard to the patients having to provide clothing, etc., and to pay for their washing, by stating that should any of the cases be too poor to undertake either outlay, it would not disqualify them for admission? The public are most generous in sending clothing to this hospital, thus enabling us to assist such cases, and the hospital has two Samaritan Funds out of which the washing bills of the necessitous can be paid.

### NURSING LECTURES.

Miss E. L. C. Eden writes from The Grange, Kingston, Taunton: So much has appeared in your columns lately on the danger of nursing lectures to the public that I am moved to say something on the other side, if you will allow me. I may mention that I have had a good deal of experience of the result of such lectures. That the amount of information assimilated is small I grant, but that the danger is great I beg leave to doubt. I firmly believe that, in course of time, great good is done by such lectures given by a competent person; but one must be content with small results at first. To answer some of the points you have raised: People do not, I think, take to nursing their friends because they have been to nursing lectures. They nurse them anyhow in certain classes, and nurse them without a glimmering of the laws of health and sanitation. That one person, as in the case you quote, opens a window and chills a patient suffering from bronchitis should not weigh down the balance of those who are killed by want of air, quack medicines, neglected bed-sores, etc. Everything depends on the lecturer, who should, from first to last, impress on his or her listeners the fearfulness and wonderfulness of human life and the danger of tampering therewith. A little knowledge is not a dangerous thing—unless it is made to take the proper place of much knowledge. The result of a good course of lectures is much more likely to make people realise the necessity of doctors and trained nurses, for "Fools rush in where angels fear to tread."

### SECTARIANISM IN HOSPITALS.

"Alpha" writes: By your criticism it is obvious that you doubt the accuracy of my letter, and by misquoting facts you will have led the public to do so also. I was not a permanent staff nurse, but a staff nurse during the latter part of my training. Doubtless it would be very inconvenient, or I might say impossible, to keep a sister's post vacant until a nurse chose to leave were she permanent on the staff. But where, as in most large training schools, nurses are leaving every month, it would be a matter of waiting only a few weeks or even days. You say "you cannot, without the most convincing evidence, believe that such a course would be pursued." I would like to ask why you published the letter from "Medicus," in which he invited nurses to give their experience, if you did not wish to insert any answers to it that were contrary, I presume, to your own views? But to cast a reflection upon it, publish it only because I gave the name of my training school, and use the offensive term "Romanist" when speaking of Catholics, savours very much of bigotry. Bigotry very quietly carried into actions I and others have experienced. Also, I was trained under the present matron, but I am perfectly certain that she was not to blame. I always found her most kind, and just, as far as she could be, but a matron does not rule supreme in the matter of promotions, at least, not in that institution.

[This letter speaks for itself, but we are glad that all nurses who belong to the Roman branch of the Catholic Church are not so prone as the writer to take offence without cause.—Ed. The Hospital.]

### Appointments.

[No charge is made for announcements under this head, and we are always glad to receive and publish appointments. The information, to insure accuracy, should be sent from the nurses themselves, and we cannot undertake to correct official announcements which may happen to be inaccurate. It is essential that in all cases the school of training should be given.]

Acland Home, Oxford.—Miss Annie Brander has been appointed theatre sister. She was trained at St. Mary (Islington) Infirmary, and has since been private nurse at the Acland Home.

Birmingham and Midland Skin Hospital.—Miss Emily Wright has been appointed sister in charge of the theatre and *x*-ray and Light Department. She was trained at the Southwark Infirmary, East Dulwich.

Brentford Isolation Hospital, Middlesex.—Miss J. E. Ives has been appointed matron. She was trained at Plaistow Fever Hospital, at the London Hospital, Whitechapel, and has since been ward sister and assistant matron at Plaistow Fever Hospital.

City of London Asylum, Dartford, Kent.—Miss Florence Raynes has been appointed assistant matron. She was trained at St. Mary (Islington) Infirmary, and has since been ward sister at the General Hospital, Gloucester, and charge nurse at the Norfolk County Asylum. She holds the certificate of the Medico-Psychological Association.

Convalescent Home, Crown Hill, Devonshire.—Miss Isabel Driver has been appointed matron. She was trained at Wandsworth and Clapham Infirmary and Flaybrick Hill Hospital, Birkenhead, where she was subsequently charge nurse. She has since been Queen's nurse, has been attached for six years to the Mentone Nursing Institute, and holiday sister in several hospitals during the summer months.

Fermanagh County Hospital.—Miss Cara Mann has been appointed staff nurse. She was trained at Isleworth Infirmary, London.

Hornsey Borough Isolation Hospital.—Miss Margaret Aisthorpe has been appointed charge nurse. She was trained at St. George's Infirmary, Fulham, and the Borough

Hospital, Southend-on-Sea. She has since been staff nurse at the Miller Hospital, Greenwich, sister at Southwark Infirmary, and has done holiday duty at Montague Hospital, Mexborough, York.

Hospital for Incurable Children, Northcourt, Hampstead.—Miss Rannie has been appointed staff nurse. She was trained at the Great Northern Central Hospital, Holloway Road, London.

Linacre Lane Hospital, Bootle.—Miss Rita de Abaitua has been appointed sister. She was trained at the Mill Road Infirmary, Liverpool, and has since been sister at the Rotherhithe Infirmary, London. She has also done private nursing in Birkenhead and in Wallasey, Cheshire.

Passmore Edwards Cottage Hospital, Liskeard.—Miss Emily Amelia Hibberd has been appointed matron. She was trained at St. George's Hospital, London, and has since been at the Salop Infirmary, Shrewsbury; St. Mary Abbott's Kensington Infirmary; Braintree and Booking Cottage Hospital; the Dartmouth Cottage Hospital; the Penrith Jubilee Cottage Hospital, and other institutions.

Royal Albert Asylum, Lancaster.—Miss Marie Campbell has been appointed matron of the Brenton House Branch of the Royal Albert Asylum for Training the Feeble-minded, Lancaster. She was formerly matron of the Victoria Nursing Home at Shanghai.

Royal Hospital, Chelsea.—Miss Ada Schofield Simpson has been appointed night sister. She was trained at the Manchester Royal Infirmary, where she was afterwards staff nurse. She has since been charge nurse at the Belgrave Hospital, Clapham Road, London.

St. Pancras Infirmary.—Miss Gertrude Arnold has been appointed sister. She was trained at Bethnal Green Infirmary, where she was afterwards staff nurse. She has since been staff nurse at St. Peter's Hospital, Covent Garden, London, and charge nurse at the Isolation Hospital, Surbiton.

Shirley Warren Infirmary, Southampton.—Miss Florence Hughes has been appointed sister. She was trained at St. Mary (Islington) Infirmary, and has since been staff nurse at Camberwell Infirmary.

### Presentations.

Ashton Infirmary.—Last week the nursing staff of the Ashton District Infirmary presented Miss Clifton, the late matron, with a silver rose bowl as a small token of their appreciation of her unfailing kindness and efforts on their behalf during her residence at the institution.

### Novelties for Nurses.

(By our Shopping Correspondent.)

### AUTUMN MATERIALS.

In preparation for the hard winter that the weather prophets assure us is bound to follow such an abnormally hot summer, it would be wise to think of obtaining a supply of sundry tweed or serge garments. Messrs. Egerton Burnett's Royal Serge Warehouse, Wellington, Som., supplies all that the most exacting taste can desire in these directions. They have a great variety of tweeds (which are to be so fashionable this year); serges of every degree of thickness, and useful hopsack or cloth materials. The "Mendip" for cycling and general wear, at 1s. 4½d. per yard, 52 inch wide, is a real bargain. There are some very smart tartans for blouses at 2s. 11½d. per yard, and well-wearing velveteens at 2s. 6d. per yard, either corded or plain. As to materials for nurses' uniforms, etc., there is a special illustrated price-list devoted to these alone, and since it also contains all necessary instruction as to self-measurement for cloaks, etc., would-be buyers cannot do better than possess themselves of one. The tailoring department is a great feature of the Royal Serge Warehouse, and nurses' cloaks are made to measure in good, all-wool serge from 24s. 6d., or with capes from 28s. 6d. Nurses' cycling skirts are made from 11s., and cycling costumes from 27s. 3d. The collars at 6¾d. each and the cuffs at 9¾d. a pair, of pure linen, are serviceable, neat and cheap. There is also a large choice of washing dress materials—all fast colours—from 7d. a yard.

### MR. MOORE'S SPECIALITIES IN NURSES' WEAR.

In writing a notice of the excellent wear which Mr. Moore, of the Albion Belfast Linen Warehouse, Albion Square, Leeds, has to offer nurses, I cannot forbear to comment on the nurses' caps. They are quite the daintiest and prettiest articles of the kind I have ever seen, especially the dainty little spotted muslin caps with their pretty frills. They are as inexpensive as they are dainty. Another novelty is one of mixed spotted muslin and plain lawn and lace, and a muslin Puritan cap, with the revers edged with lace. The nurse who cannot satisfy herself from Mr. Moore's collection of caps must indeed be difficult to please. Turning to more important matters, I have pleasure in drawing attention to the sensible well-shaped and well-made nurses' cloaks. Any shape can be secured in grey, blue, or black. The cloth cloak is made with a detachable plain cape. An excellent serge is made as plain as possible in one, and another is made also in one, with the fullness on the shoulders gathered into the plain yoke. The most expensive of these cloaks is only 24s., and the really handsome cloth cloak is only 21s. I have inspected the neat and serviceable nurses' bonnets with admiration. In blue or black they are made in fine straw, trimmed with good velvet of either colour. They are trimmed with a plain coronet-shaped wide band of velvet, relieved from its stiffness by softly folded velvet between it and the crown, with a full ruche of velvet at the edge and a full bow of the same behind resting against the crown, and with a large full bow only in the front. The quality is so good that it would be impossible to procure anything better at the price, which commences at 7s. 11d. The aprons are supplied in the excellent materials which are to be expected from a Belfast house. The finest of these aprons is made of nainsook. This is beautifully finished, and is only 2s. 3d.; others gradually ascend the scale of heaviness and afford an opportunity to secure exactly the quality to one's taste. Collars, cuffs, over-sleeves, and belts are made in good materials and carefully finished. A novelty amongst the excellent selection is a fascinating little hanging shamrock pincushion in green velvet and silk. Most nurses are familiar with the Red Cross pincushion in leather which is so attractive.

### COTTON WOOL FOR UNDERCLOTHING.

There are many people who find flannel and all woollen materials exceedingly irritating to the skin. To such and to many others Dr. Lahmann's reform cotton wool underclothing will be welcome. Dr. Lahmann is the proprietor and resident head physician of the Lahmann Sanatorium, near Dresden. He has found that woollen underclothing causes a paramagnetic irritation of the skin, the sensibility of which it gradually blunts, and he maintains that wearers of wool next the skin become very susceptible to changes of weather. It is claimed for Dr. Lahmann's fabric that it is warm in winter, cool in summer, and non-irritating. It is soft as silk; it washes well, and is inexpensive. Garments of all sorts for underwear for men, women, and children may be had from the agency, 15 Fore Street, London, E.C.

### A BEAUTIFUL WINCEY.

Macgregor's soft dressed wincey, which is made of two-thirds wool, is a delightful material for children's dresses, blouses, dressing-gowns, and underwear. The manufacturers state that it will not shrink. It is thirty inches to forty-four inches wide, and is made in medium, stout, fine, and extra fine thickness. The medium texture is quite fine enough for ordinary use. Most of the qualities can be had in all colours and others in striped or coloured mixtures. It is supplied by Messrs. Greensmith and Downes, Edinburgh.

### A Book and its Story.

### A ROMANCE OF OLD GUERNSEY.*

Mr. Austin Clare's romance carries the reader back a century or more ago, and deals with a pathetic incident which has passed into a tradition, connected with a daughter of the island of Guernsey, and a little cove, that guards its southern coast—commonly known as La petite Porte—The Little Gate. This little cove, which still nestles under the granite rocks round which the waves foam, as the tide rolls in over its sandy floor, and in springtime has its fringe of primroses and hawthorne and overhanging ivy to give an added charm to its seclusion, became to the woman of the story "no mere little gate, that might lead her anywhere, but the Little Gate of Tears. Instead of an entrance into Paradise it became the portal of Purgatory." Descendants of the heroine's family are still living in the island, we are told. And the author has, naturally, changed the names of the actors in the drama, and used an artist's license, in presenting his romance, which is most skilfully and sympathetically rendered. The word painting of the scenery is so vivid that the places described live again to those who know and love them, and to strangers it will act as a charm to draw them to Guernsey.

The old house at the Varclin, the home of Denise Tourtel, as described, is a typical island country house, of which fewer remain now than at the date of the story. "It was an old house, built of solid blocks of hewn grey granite, and roofed with red tiles purpled and browned by time and weather. . . . The doors, solid oak, both of them, were set under round beaded Norman arches, which might have done justice to a church or castle. The windows, of no architectural pretension, were set four square in the substantial walls, latticed with lozenges of greenish glass, the frames painted white. . . They reminded one, somehow, of the eyes of a matron of good standing and high character, looking out upon the world from behind a pair of gold-rimmed glasses, but showing nothing of what was going on within." The living-room of the house, with its old-time air and family relics, is also described : "It was a long, low room, oak-beamed, and panelled. There was the great open fireplace usually found in Guernsey farm kitchens, with its *pot-au-feu* hanging by a chain from a transverse bar fixed in the wide chimney over a small red fire. There were the carved-oak armchairs for the master and mistress, and the usual articles depending from the beams. . . . There were also shelves across the beams for keeping such things as cheeses, onions, and fruit. You had to be a person of low stature to avoid hitting your head against this store-house in the ceiling. But then, in common with most of the Guerneseyais, the members of the Varclin household were not tall." The house looked towards the beautiful Fermain Bay, and stood on a flagged terrace above the garden, over which the south wind blew. It was stocked with carnations, gillyflowers, clumps of crimson and pink peonies, and plots of sweet herbs of various sorts. What more fitting setting can be found for the heroine whose personality is as romantic as the situation of her home? Fair to look upon, wayward, and passionate, with moods as varying as the ocean itself, and as little understood, for her real nature lay deep beneath a smiling, gentle surface. "And what shall we say of Denise Tourtel herself, the only child of the house? To say she was beautiful would be to state a simple fact, a bare truth, as when one says a rose is beautiful, without detail or classification. . . . But her beauty was of a very rare and special order. If I were asked to compare her with one of the many beautiful roses which grew in that old garden at Varclin, it would be with the one that they call La France—a rose of an exquisite shape and perfume, whose creamy petals are touched by a faint pink that makes one think of the earliest flush of dawn, and whose heart is a nest of gold." Denise, as one of the acknowledged belles of her class, has many admirers, and one devoted, but importunate lover, Jéremie Le Clerc, who is a clerk in her father's store at St. Peter's Port. "He was now a man of thirty years. . . . He had been her comrade and slave as a child. He was her lover and worshipper now that she had grown up to be the fairest maiden in all the Channel Islands." It is an evening in June and Le Clerc and Denise are sitting together on the stone steps of the terrace looking towards the bay. Denise is shelling beans; a basket is by her side, and she holds in her lap the yellow earthenware bowl into which the beans drop when freed from their pods. "She wore a white cotton sun bonnet (it had fallen back from her pretty fair hair), a white apron, and a big white collar. Her little feet were cased in wooden sabots. . . . Her eyes—blue as periwinkles could you have seen them—were demurely downcast on her hands, from which the broad, white beans kept popping into the bowl as she cracked the downy pods. The long, black eyelashes rested upon a cheek soft and tenderly tinted . . . which never grew a whit more rosy for all the love speeches her companion was pouring into her ear. To his avowal that he has something important to tell her, she replies with a light laugh, in the old Norman tongue, common to them both that; "it seems to me that what you have to say is always more or less important—in your own opinion at least." Jéremie swears that no other lover shall ever claim Denise as his own. The reader is led from scenes of rustic gaiety and homely hospitality to others of tragic intensity which follow on the arrival of the handsome Breton sailor, when he finds Denise dressed for the part of the mute Queen in the Guernsey household festival on Midsummer's Eve. Many other ancient customs survived in the Island at that date, among which that known as the Chevauchée de Saint Michel was among the more picturesque. It was not unlike the English ceremony of "beating the bounds." "On a certain day in the year, usually about the month of June, there was a meeting of the Prévôts, the Vavasseurs, and other dignitaries belonging to the ancient Court of St. Michel de la Valle. These, after conferring and breakfasting together, rode gaily forth, each attended by his *pion*, or runner, to inspect the condition of the island roads. It was an occasion of much jollity and festivity, and cost as much as would have kept up the highways—such as they were before Governor Doyle made the present good roads, now over sixty years ago—for many a long day. But what of that when a day's enjoyment was at stake? Our forefathers were less utilitarian than we are, and far less inclined to put the cynical question, 'Cui bono?'" The book has folk songs, and proverbs, peculiar to Guernsey, which add to the charm of a picturesque romance, lacking in no detail necessary to make it both unique and original.

* "The Little Gate of Tears." By Austin Clare. (John Long. 6s.)

### Notes and Queries.

### REGULATIONS.

**The Editor is always willing to answer in this column, without any fee, all reasonable questions, as soon as possible.**

**But the following rules must be carefully observed.**

1. **Every communication must be accompanied by the name and address of the writer.**
2. **The question must always bear upon nursing, directly or indirectly.**

**If an answer is required by letter a fee of half-a-crown must be enclosed with the note containing the inquiry.**

*Italian Hospital.*

(8) Where is the Italian Hospital, and would a knowledge of Italian be of advantage to a probationer?—*Roma.*

The Italian Hospital is in Queen Square, Bloomsbury. Lay probationers are not accepted. The nursing is performed by Sisters of Charity.

*Massage.*

(9) Where can I obtain training in massage?—*Adelaide.*

At the National Hospital for the Paralysed and Epileptic, Queen Square, Bloomsbury; or write to the Incorporated Society of Trained Masseuses, 12 Buckingham Street, Strand, W.C.

Where can I train in massage and secure a medical certificate?—*Brighton.*

See answer to *Adelaide.*

*District Nurse.*

(10) I am a private nurse holding the C.M.B. certificate. Am I likely to secure a post as district nurse after undergoing a few months' training?—*H. R. F.*

Yes. Write to the General Superintendent, Queen Victoria's Jubilee Institute for Nursing, 120 Victoria Street, S.W.

*Poor-law Nursing.*

(11) Do union infirmaries receive probationers of 30 years of age?—*Harbour.*

This age is in accordance with the rules of a large number of the general hospitals and the Union Infirmaries.

*San Remo.*

(12) Will you give me the address of an English nurses' home in San Remo?—*M. E. T.*

Institute for Trained English Nurses, Sunny Bank, San Remo.

*Male Nursing.*

(13) I wish to become a fully-qualified male nurse? How can I obtain training?

Write to the National Hospital for the Paralysed and Epileptic, Green Square, Bloomsbury, where you can be trained for two years, receiving a certificate at the end of that time. You can also be trained as an orderly under the Army Nursing Sisters.

*Uniform.*

(14) Is it correct for a masseuse who is not a trained nurse to wear a nurse's uniform?—*D. L.*

It is certainly sailing under false colours, and in bad taste to wear a nurse's uniform without the training which it implies.

*Johannesburg.*

(15) Is it possible for a trained nurse to get work in Johannesburg?—*Plymouth.*

It is possible, but the prospects are very poor. The Johannesburg Nurses' Co-operative Society employs nurses with three years' training and experience of private work, and you had better write to the matron and inquire—72 Jeppe Street.

*Maternity Pupils.*

(16) Can you tell me what Scotch fever hospitals advertised for fever-trained nurses as maternity pupils?—*St. Annes.*

If you can tell about what date the advertisement appeared we will search our columns for you.

*Crêche.*

(17) Can I be received as a probationer at a crêche until I am old enough to enter a children's hospital?—*West Croydon.*

We know of no crêche where probationers are received. Your services might be accepted gratuitously.

*Naval Nursing Service.*

(18) What is the age limit for joining Queen Alexandra's Royal Naval Nursing Service, and where should I apply?—*V. M.*

Not under 25 nor over 30 years of age. Write to The Director-General, Medical Department of the Navy, 18 Victoria Street, S.W.

*A Trained Nurse.*

(19) What is understood by a trained nurse? What is the cheapest method and the shortest time required?—*Camberwell.*

A nurse is not regarded as fully trained unless she has served three years in a recognised training school. In most hospitals no payment has to be made.

*South Africa.*

(20) What outfit should I require for nursing in South Africa three days' journey from Cape Town, and what should I want for the voyage?—*Pansy.*

Unless you more definitely specify the locality it is impossible to answer the first question. For the voyage you must provide washing garments in a liberal manner, as there is no laundry on board. You will need warm underwear and wraps, as well as cool summer clothes.

*Nurses' Club.*

(21) Can you tell me of a nurses' club in London with a library where members may refer to the latest nursing publications?—*Salopici.*

The Trained Nurses' Club, 12 Buckingham Street, Strand, has a reference library such as you desire.

*The C.M.B. Certificate.*

(22) Is it necessary to register as a midwife now that I hold the C.M.B. certificate?—*Nurse.*

The certificate of the Central Midwives Board constitutes an authority to practise.

*Poor-law Nursing.*

(23) Is the Steyning Union Infirmary a recognised training school for nurses?—*E. W.*

Steyning Union Workhouse is a recognised training school, as lectures are delivered by the medical officers and others, and a three years' training with certificate is given.

*London Hospital.*

(24) I wish to become a probationer in the London Hospital. What must I do?—*B. M.*

Write to the Matron, the London Hospital, Whitechapel, London, E.

*Maternity Nursing.*

(25) Is there any home where maternity nurses are employed as district nurses?—*Rushholm.*

Write to the Maternity Charity and District Nurses' Home, Plaistow, E.

*Annuity Fund.*

(26) Where can I find out about the Princess Christian Trained Nurses' Annuity Fund, and are maternity nurses accepted?—*Manchester.*

Write to the Honorary Secretary of the Trained Nurses' Annuity Fund, 73 Cheapside, E.C. Princess Christian is the president.

*Epileptic.*

(27) Can you tell me of a home where an epileptic girl can be admitted free?—*Rose.*

The National Society for the Employment of Epileptics might be able to help you. But it is very difficult to secure a free home for epileptics.

*School for the Blind.*

(28) How can I secure a post as a teacher in a blind school, or as nurse to imbeciles?—*Inquirer.*

You might write to the London Society for Teaching the Blind to Read, 10 Upper Avenue, Hampstead, N., and for imbeciles to the Royal Albert Asylum and Training School for the Feeble-minded, Lancaster Moor, Lancaster.

*Surgical Books.*

(29) Can you tell me of an up-to-date book for the use of surgical nurses on preparing and assisting at operations, and one on surgical work?—*Hove.*

You will find the following useful: "On Preparation for Operation in Private Houses," 6d.; "Surgical Ward Work," 2s. 6d.; "A List of Surgical Instruments and Appliances," 1s. 8d. post free, in which the instruments are arranged as required in each operation; and "Surgical Bandaging and Dressings," 2s. All these can be procured at the Scientific Press, 28 Southampton Street, Strand, W.C.

*Abbreviations.*

(30) Where can I learn the abbreviations in medical prescriptions? *Naina.*

Get the "Nurses' Pronouncing Dictionary," 2s. 4d., from the Scientific Press, 28 Southampton Street, Strand, W.C.

**Handbooks for Nurses.**

| | Post Free. |
|---|---|
| "How to Become a Nurse: How and Where to Train." | 2s. 4d. |
| "Nursing: its Theory and Practice." (Lewis.) ... | 3s. 6d. |
| "Nurses' Pronouncing Dictionary of Medical Terms." | 2s. 6d. |
| "Complete Handbook of Midwifery." (Watson.) ... | 6s. 4d. |
| "Preparation for Operation in Private Houses." ... | 0s. 6d. |

Of all booksellers or of The Scientific Press, Limited, 28 & 29 Southampton Street, Strand, London, W.C.

### Cottage Hospital Work.

It is now eighteen months since, desiring a change, I advertised in The Hospital for a post as matron.

From several replies received I selected two for consideration, and a final decision found me three weeks later in charge of a cottage hospital in a small town situated in the midst of some of the finest scenery in the British Isles.

My little hospital is a "cottage" hospital in reality as well as in name, for it is a small house and an adjoining cottage thrown together and adapted to its present use.

It is very cosy and extremely home-like, but, as may be imagined, it is in some ways more difficult to work than one built on modern lines. We have seven beds and a cot, distributed over three wards; but, having no operation-room, we are obliged to prepare a room each time an operation is performed, and this entails extra labour. As regards appliances, means of sterilising, etc., we are fairly up to date. The staff consists of nurse-matron, probationer, and a maid, and, as a rule, we are quite busy. Occasionally we may have slack times, but the admission of a case of serious illness, or of one demanding immediate operation, is sufficient to keep us busy for days. When I first came I despaired of the possibility of efficient nursing being done without the help of a night nurse; but it is surprising to those who have not had practical experience in the work of cottage hospitals how well the cases do in these small places without one. As a rule, each new patient is not left for the first night after admission; but after that, if not seriously ill, I arrange that they shall be visited three or more times during the night. Also, within reach of each bed is an electric bell which rings into the matron's bedroom, so that assistance may be summoned at any time. No patient who is for any reason unable to ring is left without attendance, as I am fully empowered by my committee to engage the services of a trained nurse from a private nursing institution whenever I may deem it necessary. Theoretically, the decision rests with the doctor, but in practice I find that so long as the case is adequately nursed, the doctor does not trouble himself as to who has done the nursing.

Sometimes—though we are never without some patients—we are less busy than at others. Then I seize the opportunity to put "my house in order"; I organise and carry through a "spring clean," tidy cupboards, mend linen, make curtains, replenish dressings, stores, etc. We can generally find plenty to do, but we are very happy and contented, and I can quite understand why the desire to become matron of a cottage hospital is so general amongst nurses and that there are so many applicants for every post advertised.

My probationer and maid both came when I did. The committee pay the probationer a small salary, which is slightly increased the second year. She is able to learn a good deal and is very useful, but we make no pretence at "training" her in the full sense of the word.

As a parting word, I am quite sure that no one should undertake a post of this description who objects to work. In addition to nursing, my experience is that the matron has to do all the housekeeping, and superintendence of the maid, probably all the cooking, and very often a good deal of probationer's work.

### To Nurses.

We invite contributions from any of our readers, and shall be glad to pay for "Notes on News from the Nursing World," "Incidents in a Nurse's Life," or for articles describing nursing experiences at home or abroad dealing with any nursing question from an original point of view, according to length. The minimum payment is 5s. Contributions on topical subjects are specially welcome. Notices of appointments, letters, entertainments, presentations, and deaths are not paid for, but we are always glad to receive them. All rejected manuscripts are returned in due course, and all payments for manuscripts used are made as early as possible after the beginning of each quarter.

### For Reading to the Sick.

### TRUST AND OBEDIENCE.

When we cannot see our way,
Let us trust and still obey;
He who bids us forward go,
Cannot fail the way to show.
Though the sea be deep and wide,
Though a passage seem denied;
Fearless let us still proceed,
Since the Lord vouchsafes to lead. *Anon.*

In confidence of Thy goodness and great mercy, O Lord, I draw near unto Thee, as a sick person to the Healer, as one hungry and thirsty to the Fountain of life, a creature to the Creator, a desolate soul to my own tender Comforter. Behold, in Thee is all whatsoever I can or ought to desire; Thou art my Salvation and my Redemption, my Hope and my Strength. Rejoice therefore this day the soul of Thy servant; for unto Thee, O Lord, have I lifted up my soul.—*Thomas à Kempis.*

Out of obedience and devotion arises an habitual faith, which makes Him, though unseen, a part of all our life. He will guide us in a sure path, though it be a rough one: though shadows hang upon it, yet He will be with us. He will bring us home at last. Through much trial it may be, and weariness, in much fear and fainting of heart, in much sadness and loneliness, in griefs that the world never knows, and under burdens that the nearest never suspect. Yet He will suffice for all. By His eye or by His voice He will guide us, if we be docile and gentle; by His staff and by His rod, if we wander or are wilful: anyhow, and by all means, He will bring us to His rest. It is prayer, meditation, and converse with God, that refreshes, restores, and renews the temper of our minds at all times, under all trials, after all conflicts with the world. By this contact with the world unseen we receive continual accesses of strength. As our day, so is our strength. Without this healing and refreshing of spirit, we become fretful, irritable, and impatient.—*H. E. Manning.*

Say to Him, "Teach me to do Thy will, for Thou art my God," and He will say unto thy soul, "Fear not; I am thy salvation." He will speak peace unto thy soul; He will set thee in the way; He will bear thee above things of sense and praise of man, and things which perish in thy grasp, and give thee, if but afar off, some glimpse of His own unfading, unsetting, unperishing brightness and bliss and love.—*E. B. P.*

Learn to be as the angel, who could descend among the miseries of Bethesda without losing his heavenly purity or his perfect happiness. Gain healing from troubled waters. Make up your mind to the prospect of sustaining a certain measure of pain and trouble in your passage through life. By the blessing of God this will prepare you for it; it will make you thoughtful and resigned without interfering with your cheerfulness.—*J. H. N.*

O Shadow in a sultry land!
We gather to Thy breast,
Whose love, enfolding like the night,
Brings quietude and rest,
Glimpse of the fairer life to be,
In foretaste here possessed.
*C. M. Packard.*

## Figures and Tables

**Figure f1:**
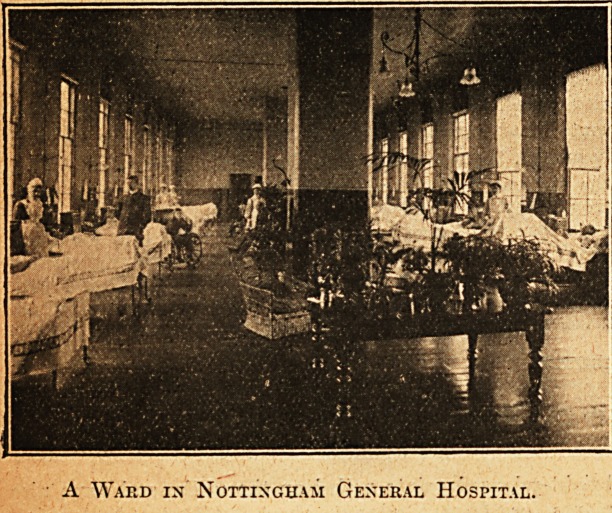


**Figure f2:**
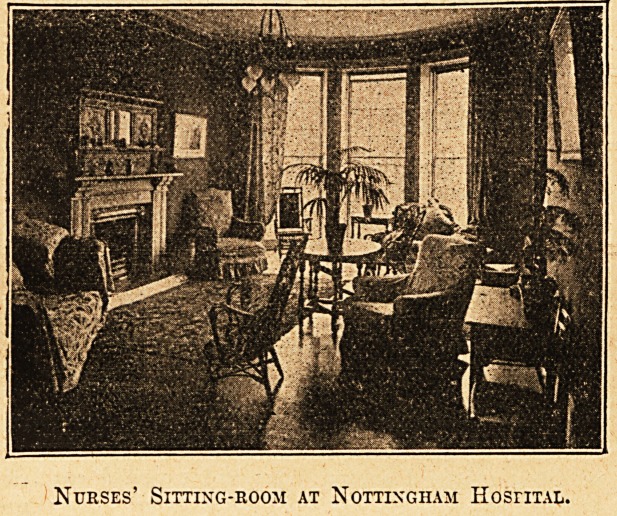


**Figure f3:**
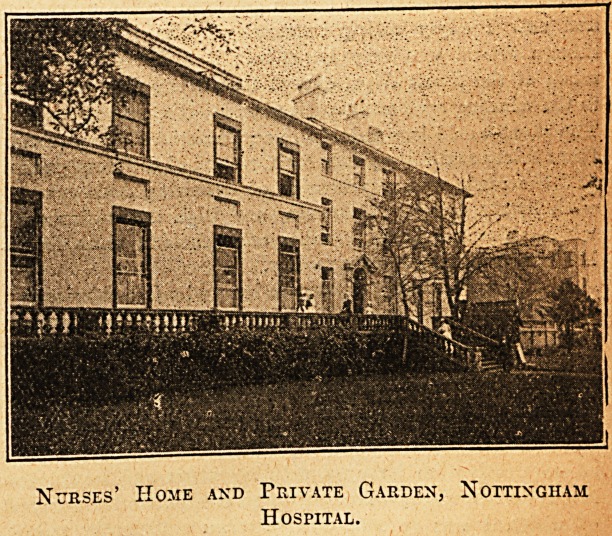


**Figure f4:**
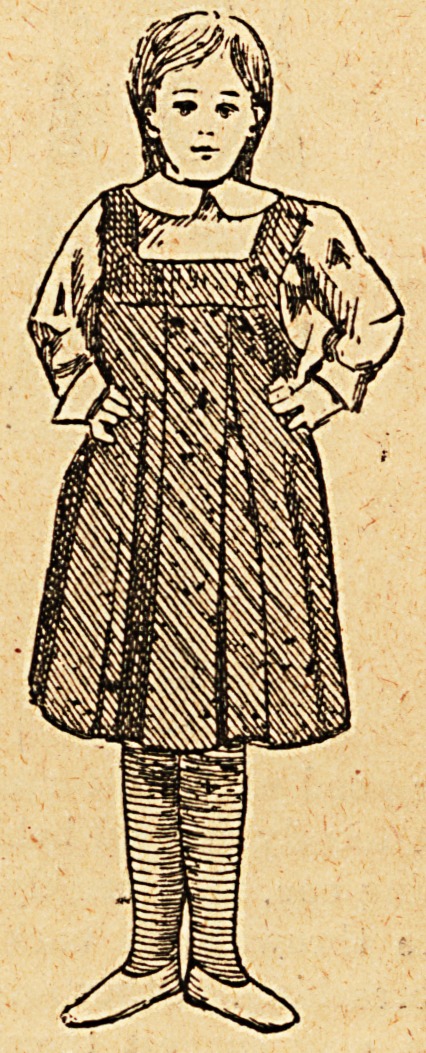


**Figure f5:**
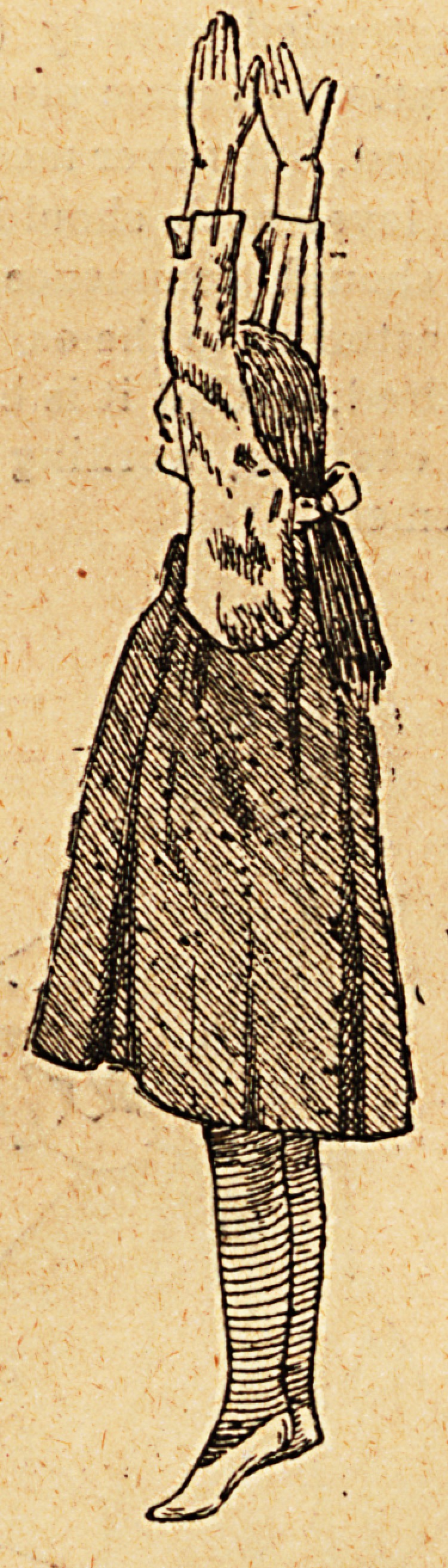


**Figure f6:**
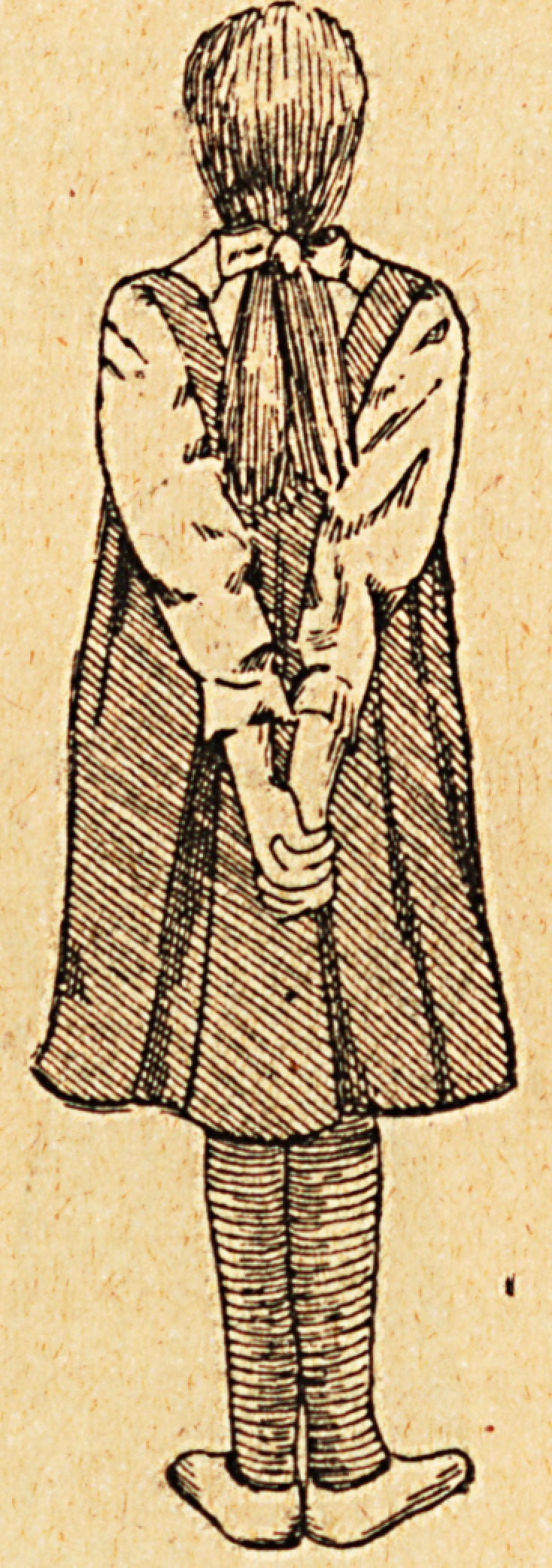


**Figure f7:**
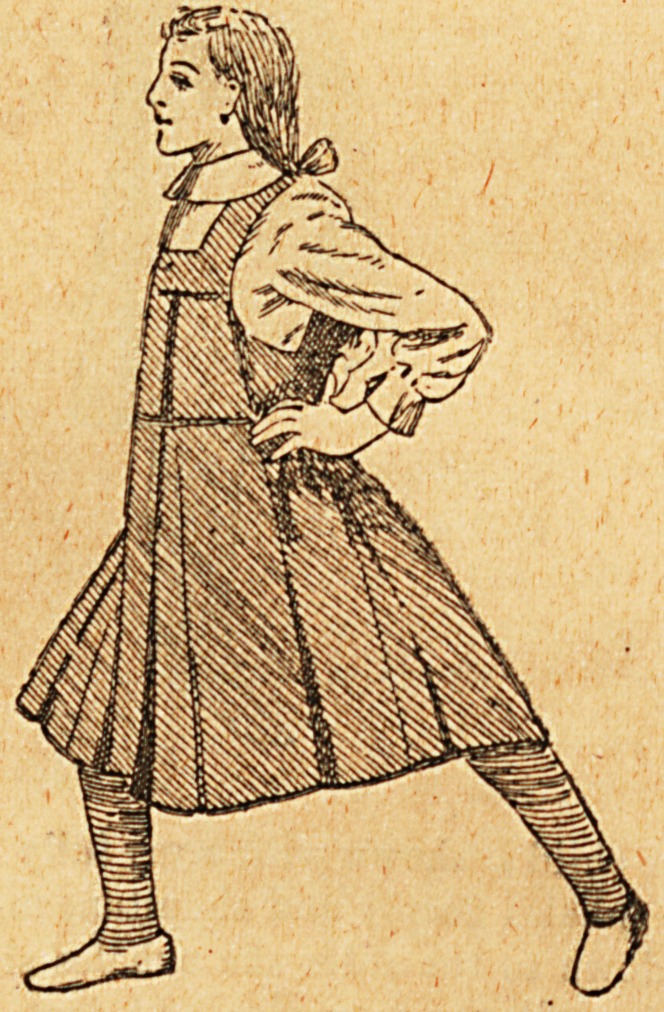


**Figure f8:**